# Molecular mechanisms of developmental pathways in neurological disorders: a pharmacological and therapeutic review

**DOI:** 10.1098/rsob.210289

**Published:** 2022-03-16

**Authors:** Niraj Kumar Jha, Wei-Chih Chen, Sanjay Kumar, Rajni Dubey, Lung-Wen Tsai, Rohan Kar, Saurabh Kumar Jha, Piyush Kumar Gupta, Ankur Sharma, Rohit Gundamaraju, Kumud Pant, Shalini Mani, Sandeep Kumar Singh, Ricardo B. Maccioni, Tirtharaj Datta, Sachin Kumar Singh, Gaurav Gupta, Parteek Prasher, Kamal Dua, Abhijit Dey, Charu Sharma, Yasir Hayat Mughal, Janne Ruokolainen, Kavindra Kumar Kesari, Shreesh Ojha

**Affiliations:** ^1^ Department of Biotechnology, School of Engineering and Technology (SET), Sharda University, Greater Noida, Uttar Pradesh 201310, India; ^2^ Department of Life Science, School of Basic Science and Research, Sharda University, Greater Noida, Uttar Pradesh 201310, India; ^3^ Division of General Surgery, Department of Surgery, Taipei Medical University Hospital, Taipei 11031, Taiwan; ^4^ Department of Medicine Research, Taipei Medical University Hospital, Taipei 11031, Taiwan; ^5^ Department of Information Technology Office, Taipei Medical University Hospital, Taipei 11031, Taiwan; ^6^ Graduate Institute of Data Science, College of Management, Taipei Medical University, Taipei 110, Taiwan; ^7^ Indian Institute of Management Ahmedabad (IIMA), Gujarat 380015, India; ^8^ ER Stress and Mucosal Immunology Laboratory, School of Health Sciences, University of Tasmania, Launceston, Tasmania 7248, Australia; ^9^ Department of Biotechnology, Graphic Era deemed to be University Dehradun Uttarakhand, 248002 Dehradun, India; ^10^ Department of Biotechnology, Jaypee Institute of Information Technology, A-10, Sector 62, Noida, Uttar Pradesh 201301, India; ^11^ Indian Scientific Education and Technology Foundation, Lucknow 226002, India; ^12^ Laboratory of Neurosciences and Functional Medicine, International Center for Biomedicine (ICC) and Faculty of Sciences, University of Chile, Santiago de Chile, Chile; ^13^ School of Pharmaceutical Sciences, Lovely Professional University, Phagwara 144411, Punjab, India; ^14^ Department of Pharmacology, School of Pharmacy, Suresh Gyan Vihar University, Mahal Road, 302017 Jagatpura, Jaipur, India; ^15^ Department of Chemistry, University of Petroleum and Energy Studies, Dehradun 248007, Uttarakhand, India; ^16^ Discipline of Pharmacy, Graduate School of Health, University of Technology Sydney, Sydney, New South Wales 2007, Australia; ^17^ Department of Life Sciences, Presidency University, 86/1 College Street, Kolkata 700073, India; ^18^ Department of Internal Medicine, College of Medicine and Health Sciences, United Arab Emirates University, PO Box 15551, Al Ain, United Arab Emirates; ^19^ Department of Applied Physics, School of Science, and; ^20^ Department of Bioproducts and Biosystems, School of Chemical Engineering, Aalto University, Espoo 00076, Finland; ^21^ Department of Pharmacology and Therapeutics, College of Medicine and Health Sciences, United Arab Emirates University, PO Box 15551, Al Ain, United Arab Emirates; ^22^ Department of Health Administration, College of Public Health and Health Informatics, Qassim University, Buraidah, Saudi Arabia

**Keywords:** Wnt/β-catenin, Notch, Sonic hedgehog, CNS, neurodegeneration, neurotherapeutics

## Abstract

Developmental signalling pathways such as Wnt/β-catenin, Notch and Sonic hedgehog play a central role in nearly all the stages of neuronal development. The term ‘embryonic’ might appear to be a misnomer to several people because these pathways are functional during the early stages of embryonic development and adulthood, albeit to a certain degree. Therefore, any aberration in these pathways or their associated components may contribute towards a detrimental outcome in the form of neurological disorders such as Alzheimer's disease, Parkinson's disease, amyotrophic lateral sclerosis and stroke. In the last decade, researchers have extensively studied these pathways to decipher disease-related interactions, which can be used as therapeutic targets to improve outcomes in patients with neurological abnormalities. However, a lot remains to be understood in this domain. Nevertheless, there is strong evidence supporting the fact that embryonic signalling is indeed a crucial mechanism as is manifested by its role in driving memory loss, motor impairments and many other processes after brain trauma. In this review, we explore the key roles of three embryonic pathways in modulating a range of homeostatic processes such as maintaining blood–brain barrier integrity, mitochondrial dynamics and neuroinflammation. In addition, we extensively investigated the effect of these pathways in driving the pathophysiology of a range of disorders such as Alzheimer's, Parkinson's and diabetic neuropathy. The concluding section of the review is dedicated to neurotherapeutics, wherein we identify and list a range of biological molecules and compounds that have shown enormous potential in improving prognosis in patients with these disorders.

## Graphical abstract

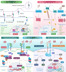

## 1. Introduction

Neurological disorders are characterized by a gradual and progressive loss of neurons, which ultimately affects the steady-state homeostasis of the human nervous system and thereby functions such as abstract thinking, movement, emotions, cognition and memory [1]. Available data suggest that nearly 2% of the global population may be affected by such detrimental outcomes [2]. Some of the common neurological disorders include Alzheimer's disease (AD), Parkinson's disease (PD), amyotrophic lateral sclerosis (ALS), multiple sclerosis (MS), stroke and diabetic neuropathy. Studies have identified several risk factors such as genetic polymorphisms, ageing, endocrine conditions, oxidative stress, inflammation, hypertension, diabetes, depression, infection, vitamin deficiencies, metabolic conditions, chemical exposure and dietary supplements, among others, that can drive the pathogenesis of these disorders [3–5]. In addition to these risk factors, a panel of signalling pathways that are crucial during normal brain functioning can also drive the pathophysiology of brain-related disorders. It is a well-known fact that signalling is indeed a key biological activity that ultimately decides the fate, phenotype and response of all cells in the human body. This is no different in the case of brain cells, and therefore signalling pathways, especially in neurological disorders, has been a subject of extensive research in the last decade. Although tremendous progress has been made on that front, a lot remains to be deciphered.

**Figure 1.**
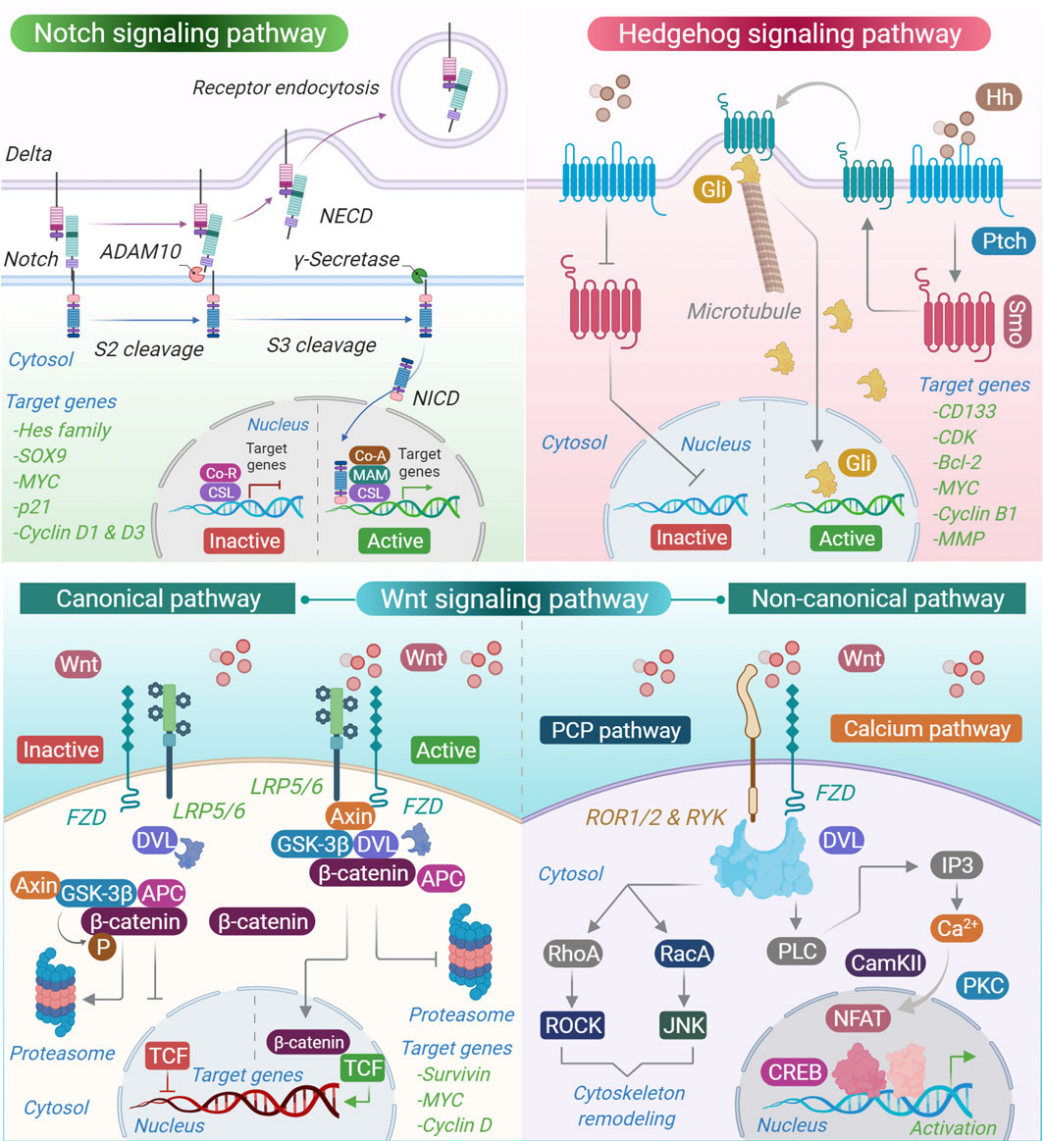
Developmental signalling pathways and associated activation process.

Embryonic signalling pathways or developmental pathways (figure 1) are highly conserved cellular activities in vertebrates. As the name suggests, these pathways are mostly active during embryonic development, especially within the neural tube and skeleton, and are mostly dormant or have low activity in adult tissues. During embryogenesis, they take centre stage within the central nervous system (CNS) and define the fate of neural progenitor cells (NPCs) and their neuronal and glial progenies, thereby facilitating normal brain development and operation. Emerging reports suggest that embryonic signalling plays a key role in the formation and plasticity of neuronal circuits in the hippocampal region of the brain, which is fundamental to learning and memory. In addition, these pathways can act as pleiotropic factors and facilitate several other developmental processes such as embryonic patterning, brain development, proliferation, specification and axonal targeting in the forebrain, hindbrain and spinal cord. As mentioned, these pathways remain operational in adults, albeit to a certain degree, and modulate normal brain homeostasis and adult neurogenesis [6,7] Thus, any aberration or deregulation in these pathways or their associated components may lead to detrimental outcomes that can drive the pathogenesis of several neurological disorders. Nevertheless, despite the criticality of these pathways, concrete and clear information pertaining to their activity and/or interactions in neurological disorders is largely elusive, and hence a subject of continued research. Therefore, detailed insight into the individual pathways is essential to identify the disease-critical interactions of these pathways, which in turn may lay a platform for future therapeutics. Essentially, there are three embryonic signalling pathways, namely Notch, Sonic hedgehog (Shh) and Wnt/β-catenin. In this review, we explore the functional significance and disease critical interactions of these pathways in a range of neurological disorders. In addition, we investigated the possibility of using biomolecules and/or molecular compounds that have shown tremendous promise in targeting these abnormal interactions, which could lay a platform for novel neurotherapeutics.

## 2. Developmental signalling pathways: a general overview

### 2.1. Notch signalling pathway

The Notch pathway is widely studied as a key developmental pathway; however, it is highly active and operational in the adult brain. Notch signalling modulates several key aspects of neurogenesis such as morphology, migration, synaptic plasticity, maintenance of mature and immature neurons, radial glia maintenance, neurogenesis and dendrite development. Most importantly, Notch drives the activity of neural stem cells (NSCs) by mediating the epigenetic remodelling of glial fibrillary acidic protein that in turn maintains the competence of NSC [8–10]. Studies have suggested that the Notch pathway may be crucial in long-term memory formation, as well as memory loss following severe neurodegeneration [11]. Similar to other embryonic pathways, the juxtracrine Notch signalling pathway is highly sustained across vertebrates. Notch has four homologues (Notch 1, 2, 3 and 4) and two sets of ligands belonging to the Jagged (Jag-1 and Jag-2) and Delta (Delta-like-1, 3 and 4) families. All Notch receptors are non-covalently bound heterodimeric, single-pass transmembrane proteins. The Notch pathway is activated by a ligand–receptor interaction-induced conformational change in the Notch receptor architecture, which exposes the cleavage sites in the extracellular domain of the receptor. Thereafter, a disintegrin and metalloprotease 10/17 (ADAM 10/17) mediates the first cleavage (S1 cleavage), resulting in the release of the residual Notch C-terminal (NEXT) fragment. The NEXT fragment is then subjected to a second cleavage (S2 cleavage) mediated by the enzyme γ-secretase (gamma-secretase). The S2 cleavage is extremely crucial because it releases the nucleus-bound fragment, the Notch intracellular domain (NICD). Thereafter, the NICD translocates into the nucleus and forms a transcriptionally active complex along with co-activators belonging to the CBF-1/Su(H)/Lag-1 (CSL) and mastermind-like (MAML) family of proteins. This complex regulates the transcription of an array of Notch downstream target genes, namely Hes family bHLH transcription factor 1 (Hes-1) and Hes-related family bHLH transcription factor with YRPW motif (Hey). Both Hes-1 and Hey are the negative regulators of transcription [12–18].

### 2.2. Shh signalling pathway

The Shh pathway is strongly associated with the development of the neural tube, patterning of the ventral structures and ventral forebrain, neuronal differentiation, proliferation and survival of ventral progenitors, specification of ventral neurons, midbrain dopaminergic differentiation, cerebellar neuronal precursor proliferation and patterning of the developing thalamus. In humans, the subventricular zone (SVZ) of the brain is one of the two sites where neurogenesis occurs in adults and Shh signals set the tone for the same [19–22]. Similar to Notch, Shh along with the epidermal growth factor (EGF) regulates the activity of NSCs during embryogenesis and in adult brains. Several studies have reported that Shh transactivates the EGF receptor (EGFR), resulting in ERK1/2 signalling in NSCs, suggesting a collaborative activity mostly during the later stages of NSC proliferation [23]. The Shh signalling pathway primarily involves a 12-transmembrane protein, protein patched homologue 1 (Ptch-1) and an array of ligands, namely Shh, Desert Hedgehog and Indian Hedgehog. In organisms such as zebrafish, Shh has three additional ligand homologues, namely Qiqihar Hedgehog, Echidna Hedgehog and Tiggy-winkle Hedgehog [24–26]. Shh is the most broadly expressed and widely investigated homologue in mammalian systems. The interaction of Shh with Ptch-1, which is expressed on the membrane of receptive cells, activates the Shh signalling pathway. Incidentally, in the absence of Shh activation, PTCH1 behaves as a constitutive repressor of another Hh transmembrane receptor, smoothened (Smo). Smo is a member of the G-protein coupled receptor (GPCR) family. Ptch-1 and Smo act as transducers of Shh signals. Shh-induced Smo activation drives several cell fate decisions through the activation of an array of zinc finger transcription factors such as cubitus interruptus (Ci) in *Drosophila* and GLI family zinc finger (Gli) in mammals. Ci can be phosphorylated by a panel of kinases such as protein kinase A (PKA), glycogen synthase kinase-3β (GSK-3β) and casein kinase 1α (CK1α). Further, three Gli proteins, namely Gli-1, Gli-2 and Gli-3, operate as downstream effectors of the Shh pathway in mammals. Gli-1 acts only as a transcription activator, whereas Gli-2 and Gli-3 act as an activator or a repressor in a context-dependent manner. The activation of the Shh signalling pathway in a cell perturbs the ratio of the Gli activator and Gli repressor, resulting in diverse cellular responses proportional to the strength and nature of the Hh signal. In addition to Ci and Gli, other components such as microtubule-associated kinesin-like protein Costal 2 (Cos2), serine–threonine kinase fused (FU) and the suppressor of fused (SUFU) form an integral part of the Shh cascade in vertebrates [24,27–29].

### 2.3. Wnt/β-catenin signalling pathway

The Wnt/β-catenin pathway is highly conserved across the metazoans and drives key cellular functions such as cell specialization, migration, adhesion and development [30]. Recent studies have highlighted the role of the Wnt/β-catenin pathway in normal brain development, especially in regulating the functions of mature neurons in the adult CNS [31,32]. In addition, the Wnt/β-catenin pathway is associated with a myriad of other processes such as neuronal maturation, axon remodelling, neuronal connectivity, migration and synaptic formation in the embryonic brain. In the adult brain, the Wnt/β-catenin pathway drives synaptic activity and behavioural plasticity [33–35]. In mammals, Wnt proteins are coded by 19 Wnt genes and are essentially lipid-modified glycosylated cysteine-rich proteins with molecular sizes ranging from 39 to 46 kDa [31,36]. The canonical Wnt signalling pathway is activated by interaction between Wnt and transmembrane pass receptor, Frizzled (Fz). The non-canonical Wnt signalling pathway is activated by proteins such as LRP5/6 that serve as one of the many co-activators of the Wnt signalling pathway. After the ligand–receptor interaction, the pathway is triggered by the localization of the protein called Dishevelled (Dvl). Thereafter, Dvl interacts with and recruits the destruction complex comprising Axin, adenomatous polyposis coli (APC), serine–threonine kinases (glycogen synthase kinase-3 (GSK-3) and casein kinase 1α (CK1α)) and protein phosphatase 2A (PP2A). The recruitment of destruction complex allows the accumulation of the Wnt downstream effector β-catenin, resulting in increased cytosolic accumulation of β-catenin. Eventually, β-catenin is translocated into the nucleus. The interaction of β-catenin with the TCF/LEF family of transcription factors in the nucleus leads to the activation of a panel of Wnt downstream target genes [37–40].

## 3. Developmental signalling pathways and ageing brain

Brain shrinkage and changes in the brain at all levels from molecules to morphology are associated with increasing age. The major cellular and molecular mechanisms responsible for brain ageing are mitochondrial dysfunctions, impaired molecular disposal, impaired DNA repair, aberrant neuronal network activity, oxidative damage, deregulated neuronal calcium homeostasis, impaired adaptive stress response signalling, stem cell exhaustion, glial cell activation and inflammation. In addition to other signalling pathways, the embryonic signalling pathways are crucial for the development and proper functioning of the brain. The embryonic signalling pathway crosstalk is complex and highly required for the molecular regulation of homeostasis and adaptation of neuronal cells. The alterations in these pathways render the ageing brain vulnerable to various neurological disorders.

The Wnt signalling pathway is an important pathway at the synapse and is required for synaptic plasticity and maintenance in the adult brain. Increasing studies have recommended that synaptic signalling is compromised in the ageing brain, leading to synaptic failure. The synaptic strength and functions normally reduce with age, making these synapses susceptible to different toxic molecules including amyloid beta (Aß). Hence, we observe changes in the Wnt signalling pathway and other signalling pathways that are important for synapse integrity in an ageing brain [41–43].

Several studies have suggested that Wnt signalling is reduced in the aged human brain. Studies have further revealed that Wnt ligands (Wnt-2b, Wnt-6 and Wnt-7a), as well as frizzled receptors (Fzd-2 and Fzd-3), are downregulated in the aged human brain [44]. A study conducted by Inestrosa *et al.* [45] revealed that soluble endogenous inhibitors of the Wnt signalling pathway increase in an age-dependent manner in both the hippocampus and cortex of *Octodon degus*. In addition, they reported that age-related Wnt signalling defects were recovered by andrographolide treatment in *O. degus* [45].

A recent study proposed that defective Notch signalling may play a critical role in the pathophysiology of neurodegenerative diseases. Furthermore, studies have revealed that Notch signalling in neurons, glia and NSCs may be intricate in pathological alterations that occur in age-related disorders. Interestingly, animal model-based studies have highlighted the therapeutical potential of different agents targeting the Notch signalling, in the case of age-related central nervous system (CNS) disorders. Also, the Notch signalling pathway is evolutionarily conserved and found to be important for vascular development and function. Age-related alterations in Notch signalling may elicit neurovascular dysfunction, leading to the progression of neurodegenerative diseases [46].

The Notch and Wnt pathways are known to interact with each other. The communication and equilibrium between these pathways may be interrupted in ageing individuals and ageing-related diseases. For instance, stable interaction between these pathways is necessary for the renewal of adult skeletal muscles and angiogenesis; however, progressing age may disrupt such connections and limit regenerative capacity.

In addition to defective Wnt and Notch signalling, defective Shh signalling may play a role in the pathogenesis of age-related neurological disorders. In the recent past, the Shh signalling pathway was reported to play a role in neurogenesis, anti-inflammatory and antioxidant pathways, and autophagy [47]. Hence, the Shh signalling pathway may be a significant modulator in age-related neurological diseases.

## 4. Developmental signalling pathways involved in maintaining blood–brain barrier integrity

**Figure 2.**
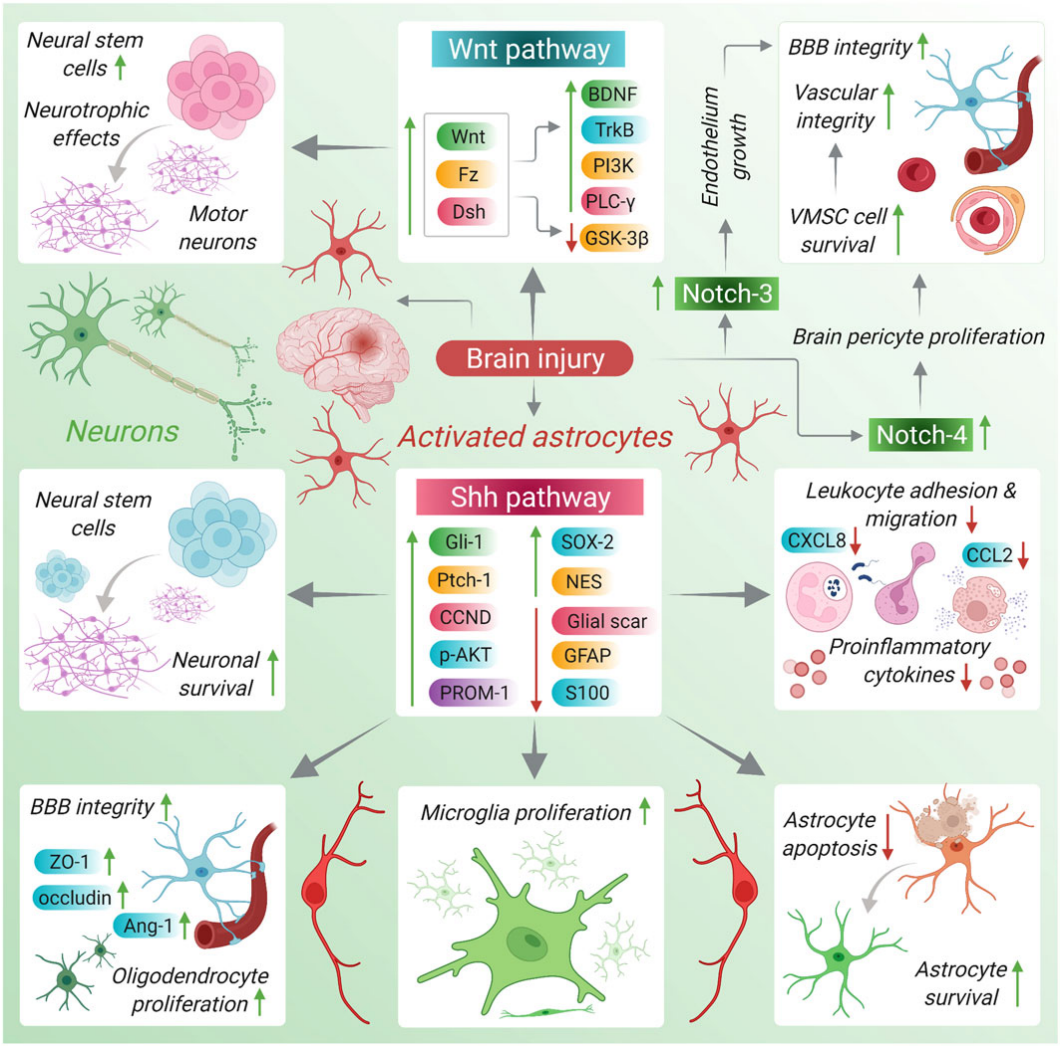
Disease–critical interactions in response to brain trauma, injury or disorder.

The blood–brain barrier (BBB) is a highly selective and permeable barrier that separates the circulating blood from the extracellular fluid of the brain to regulate the CNS microenvironment [48]. The BBB is made up of endothelial cells (ECs), astroglia, pericytes, perivascular macrophages and basal membrane. The ECs, through tight junctions (TJs) and basal lamina, maintain BBB integrity under normal physiological conditions. The structural and functional integrity of the BBB is severely altered during events such as neoplasia, ischaemia, trauma, inflammation, and bacterial and viral infections. Brain traumas such as permanent ischaemia lead to the redistribution of claudin decomposition fragments, zona occludens-1 (ZO-1) and occludin from the membrane to the cytoplasm in BBB. The Notch signalling pathway is involved in maintaining blood vessel integrity and BBB stability. Several studies have reported a strong correlation between endothelial dysfunction and deregulated Notch expression [49]. For instance, the Notch-4-based activation of the Notch signalling pathway helps in maintaining the stability and growth of the mature BBB endothelium (figure 2) [49]. Studies have suggested that gamma-secretase inhibitors (GSIs) such as N-[N-(3,5-difluorophenacetyl)-1-alany1-S-phenyglycine t-butyl ester (DAPT) confer protection to the brain against permanent ischaemia-induced BBB damage by altering the Notch-4/Calpastatin homeostasis pathway in vascular ECs (VECs). In addition to VECs, brain pericytes play a role in regulating brain vascular integrity, permeability and blood flow, and therefore brain pericytes are an important component of the BBB. The deficiency of brain pericytes has been attributed to neonatal intracranial haemorrhage in human fetuses and stroke and neurodegeneration in adults. In *in vitro* studies, Notch signalling inhibition, either by GSIs or by genetic ablation of endothelial Notch, has been found to contribute to BBB impairment, as substantiated by altered localization and loss of endothelial junction molecules, decelerated transendothelial electrical resistance and augmented macromolecular permeability. Further, inflamed brain ECs (BECs) had altered activity of Notch components, as indicated by decreased expression of the downstream target genes (Hes-1 and Hes-5). Notably, barrier function was further decelerated when the Notch signalling pathway was hampered under the inflammatory state, signifying the additive outcome of the defective Notch signalling and inflammation in BECs. Conversely, the enhanced activity of inducible NICD-1 rescued the negative effect of inflammation. Interestingly, inflammation curtailed the expression of the glycosyltransferase Lunatic Fringe, a well-known positive controller of Notch glycosylation and associated signalling, and thereby contributed to the disruption of the barrier function of BECs [50]. Recent experiments conducted using zebrafish model systems have revealed that Notch-3 is expressed on the surface of pericytes and a deficiency in Notch-3 expression results in the deficiency of pericytes in the zebrafish brain. Therefore, aberrant Notch-3 expression can contribute to the improper functioning of the BBB through defective pericyte function. Moreover, conditional loss- and gain-of-function experiments have provided strong evidence that Notch-3 signalling positively regulates brain pericyte proliferation and differentiation [51]. Incidentally, Notch signalling is not restricted to VECs and brain pericytes but is fundamental for the survival of vascular smooth muscle cells (VSMCs). VSMCs maintain BBB homeostasis through Notch-3 activation-induced processes such as contraction, blood flow distribution and regulation of blood vessel diameter. Notch-3 is expressed on the surface of VSMCs in the mammalian CNS [52]. The leaky BBB in the stem cell niches of the intact and ischaemic stroke brains responds to circulating vascular endothelial growth factor (VEGF) to drive the induction of the Notch ligand (DLL4) in pericytes and ECs, and further induce significant proliferation of stem cells and neurogenesis. Notch ligand DLL4 is one of the most imperative cues in angiogenesis [53]. Overall, these studies suggest that the Notch signalling pathway plays a vital role in maintaining normal BBB homeostasis both in embryonic and adult brains.

Similar to the Notch signalling pathway, the canonical Wnt signalling pathway regulates the vascularization of the CNS and maintains BBB properties unique to the CNS vasculature in ECs. Results obtained from cell culture assays and genetic loss- and gain-of-function experiments in mice have revealed that the Gpr124 protein acts as a coactivator of the Wnt-7a- and Wnt-7b-stimulated canonical Wnt signalling pathway through the Fz receptor and Lrp coreceptor. Moreover, the Gpr124-stimulated Wnt signalling pathway operates in cooperation with the Norrin/Fz-4 signalling pathway to control CNS vascular development. This indicates that Gpr124 is a ligand-specific coactivator of the canonical Wnt signalling pathway [54]. In addition, Wnt signalling suppresses the sphingosine-1-phosphate receptor (S1pr) signalling during angiogenesis, which drives the dynamic junction formation during anastomosis. Moreover, S1pr signalling mediates BBB maturation and VE-cadherin stabilization. However, the premature activation of S1pr in the absence of Wnt signalling reduces VE-cadherin and Esama at cell–cell junctions. Interestingly, these findings reveal a crucial link between brain angiogenesis and BBB formation, and emphasize that Wnt signalling is the chief coordinator of anastomosis [55]. In neurological traumas (such as haemorrhagic stroke, seizure and CNS inflammation) defective β-catenin transcription activity results in BBB dysfunction [56]. Studies have indicated that Wnt morphogens released by neural precursor cells control BBB formation during embryogenesis. The activation of the Wnt/β-catenin pathway in mouse BECs modulates the stabilization of the endothelial TJs through transcriptional modulation of the TJ proteins. Further, the Wnt5a-mediated activation of the pathway possibly through a Par/aPKC PCP-dependant mechanism has been reported in brain microvascular endothelial cell lines (hCMEC/D3). Importantly, the activation of Wnt-5a in hCMEC/D3 cell line is completely independent of the classical Wnt/β-catenin activation, although Wnt-5a contributes significantly towards maintaining TJ integrity and endothelial apicobasal polarity. Overall, these studies suggest that the Wnt-5a/Par/aPKC PCP pathway and classical Wnt/β-catenin mediators act as key regulators of normal BBB homeostasis [57].

Few studies have investigated the effect of Shh pathway activation on BBB homeostasis and maintenance; therefore, this continues to remain a grey area. Nevertheless, reports have suggested that Shh signalling may be crucial in the maintenance of BBB integrity [58]. The astrocytes localized in the BBB secrete a variety of Shh ligands and the ECs express Shh receptors such as Ptch-1; hence, astrocytes and ECs may be involved in BBB formation and maintenance during embryonic development and adulthood, respectively. Moreover, the activation of Hh signalling renders a barrier-promoting effect and maintains an endogenous anti-inflammatory balance against CNS-directed immune attacks, as observed in several cases of multiple sclerosis (MS) [59]. Incidentally, the effect of the Shh signalling pathway is not restricted to MS. A study conducted in a cerebral ischaemia model reported that the Shh signalling pathway regulates permeability across the BBB [60]. Shh triggers angiopoietin (Ang-1) production predominantly in the astrocytes under the conditions of severe ischaemic insults. Ang-1 acts on brain microvascular ECs and upregulates the expression of proteins, ZO-1 and occludin. The upregulation of ZO-1 and occludin expression drives the repair of TJ, thereby ameliorating the symptoms of brain oedema and BBB leakage [60].

## 5. Glial cells: the platform for developmental signalling-based neuronal homeostasis

In the CNS, the neurons and glial cells are equally important; the neurons play a key role in neurotransmission and glial cells provide neuronal support and maintain the steady-state brain homeostasis. Glial cells have been attributed in a range of processes across the CNS, such as the release and uptake of neurotransmitters, pyruvate and glutathione metabolism, ion buffering, organization of BBB, production of myelin and cerebrospinal fluid (CSF). In addition, the activity of glial cells is regulated by subtle interactions between neurons and glia, wherein the embryonic pathways play the central role. The Shh signalling pathway is mostly active in the glial cells during embryogenesis and in the neural precursors during adulthood. Nevertheless, in the adult brain, Shh may be expressed by neurons and astrocytes. Several studies have suggested that the activation of the Shh signalling pathway drives several neuro-glial interactions and crosstalk. Brain trauma due to chemical reactive oxygen species (ROS and other compounds), biological (microorganism infection) and physical injuries (ischaemia and hypoxia) causes reactivation of the Shh signalling pathway in the glial cells, particularly astrocytes, of the adult brain, leading to tissue regeneration [61]. In addition, these injuries elicit contact between astrocytes and neurons through the activation of the Shh signalling pathway and associated components (figure 2). Further, the Shh signalling pathway-mediated astrocyte response to brain insults facilitates coordinated cell reaction in the brain tissue. Astrocytes participate in the brain repair processes and act as key mediators of the brain injury-specific responses. Moreover, brain insults cause astrocytes to become reactive and activate the Shh signalling pathway, which causes an enhanced expression of genes such as Ptch and Gli. Additionally, it enhances the expression of genes such as cyclin D1 protein (CCND) (cell-cycle), Nestin (NES) (cytoskeleton), SRY (sex-determining region Y)-box 2 (SOX2), Prominin 1 (PROM1) and protein kinase B (PKB) also known as AKT. On the contrary, Shh overexpression in astrocytes is correlated with decreased levels of GFAP and S100, and reduced scar formation. Most importantly, after brain insults, Shh signalling enhances the proliferation of oligodendrocytes, microglia and NG2-positive cells, and helps in neuronal survival and BBB integrity through enhanced expression of ZO-1 and occludin in microvessel ECs, thereby advocating a coordinated tissue response for brain repair [61]. Emerging evidence suggests that these post-traumatic responses are partly mediated by the activation of the Shh cascade that drives an array of cellular responses in the brain to build a neuroprotective environment primarily by reinforcing the damaged BBB [62,63].

Cerebellar development in mammals progresses under the precise spatio-temporal control of key developmental pathways such as the Wnt/β-catenin signalling pathway. Wnt/β-catenin activity is observed in the perinatal cerebellar ventricular zone (VZ), a germinal centre in the developing cerebellum that gives rise to GABAergic and glial cells. The regulation of Wnt/β-catenin signal levels is essential for the normal development of the cells arising from the cerebellar VZ during the late embryogenesis stage [64]. Several risk factors, such as genetic and hormonal background (i.e. gender and oestrogens) and endogenous and exogenous activators of Wnt/β-catenin signalling components (i.e. GSK-3β-antagonists), can stimulate astrocyte-mediated beneficial effects on brain injury or trauma. Astrocyte activation can occur through the expression of growth or neurotrophic factors, particularly Wnt-1. Astrocytes located in the ventral midbrain can facilitate neurogenesis and DAergic neurogenesis from the adult neural stem or progenitor cells by activating the Wnt/β-catenin signalling pathway. For instance, studies have shown that Wnt-1 can mediate the survival, repair and rescue of DA neurons by directing neuronal effects and by inhibiting the microglia-M1-activated phenotype. Interestingly, astrocyte-derived Wnts and the activation of the Wnt/β-catenin signalling pathway play a key role in the regulation of adult neurogenesis [65,66]. In the aged brain, the neural progenitors localized in the neurogenic areas such as the SVZ and subgranular zone (SGZ) of the hippocampus are in close contact with the astrocytes, thereby facilitating in building a directive ‘niche’ that regulates neurogenesis [67,68]. During embryonic development, Wnt-1/β-catenin signals control DAergic neurogenesis chiefly by maintaining the integrity of the generated neurogenic niche and overseeing the progression from nuclear receptor-related 1 protein-positive (Nurr1+)/TH− post-mitotic DAergic neuroprogenitors to Nurr1+/TH+ neurons [69,70]. Although the connection between glial cells and Notch signalling has not been explored, few studies have reported that Notch ligands and receptors are constitutively expressed on microglial cell surfaces in the developing brain. The Notch signalling pathway is crucial for the maintenance of the microglial population during early development, as is the case of other glial cells during normal development. In postnatal and adult rat models, the Notch signalling pathway has been attributed to microglial activation and inflammation process during neuroinflammatory diseases [49,71].

## 6. Developmental signalling and brain-derived neurotrophic factor crosstalk in the central nervous system

Brain-derived neurotrophic factor (BDNF) is a well-characterized neurotrophin that controls numerous activities in the CNS, such as neuronal differentiation, neuroprotection and synaptic plasticity. The Wnt signalling pathway plays a crucial role in maintaining BDNF expression in the brain. The activation of the Wnt/β-catenin signalling pathway upregulates BDNF expression. For instance, Wnt-3a-based signalling activation induces the expression of BDNF and several other components of the BDNF signalling pathway in neurons and glial cells [72]. Moreover, neuron activity-induced Wnt signalling can also upregulate the expression of BDNF, especially in the pain neural circuit. In principle, neuronal activity-induced BDNF gene expression is mainly regulated by the Ca^2+^/cAMP response element-binding protein (CREB) pathway; however, some studies have reported the involvement of other regulatory factors in the regulation of BDNF expression levels through interaction with the Wnt signalling pathway. In primary cortical cultures, blocking the activation of the Wnt/β-catenin signalling pathway prevents the expression of BDNF in response to the activation of the *N*-methyl-d-aspartic acid (NMDA) receptor. Wnt/β-catenin-induced BDNF expression is essential for peripheral pain-induced upregulation of BDNF expression in the mouse spine. Hence, conditional deletion of one copy of either Wntless or β-catenin is sufficient to repress the pain-induced upregulation of BDNF expression in the mouse spine [73]. Evidence suggests that BDNF can modulate the growth of human neurons possibly through crosstalk involving Wnt/β-catenin and GSK-3β. In an *in vitro* study, BDNF overexpression in human embryonic spinal cord neurons resulted in the upregulation of Wnt pathway components/factors such as TrkB, PI3K, AKT, PLC-γ, Wnt, Fz, Dsh and β-catenin and downregulation of GSK-3β (figure 2) [74]. In addition to neurons, BDNF promotes the growth of human NSCs and the Wnt/β-catenin signalling pathway is actively involved in the process. Cell culture assays have shown that transfection with pIRES2-ZsGreen1-BDNF results in increased growth of human embryonic spinal cord (hESC)-NSCs, significant upregulation of Wnt, Frizzled and Dsh expression, and reduction in the GSK-3β level. On the contrary, treatment of cultured hESC-NSCs with BDNF-siRNA reverses the phenotype. Overall, these observations suggest that BDNF signalling can affect the growth of neurons and hESC-NSCs *in vitro*, possibly through crosstalk with the Wnt signalling pathway, and GSK-3β appears to be the key link connecting these two pathways [75].

The effect of Shh cascade activation on BDNF expression has not been studied in great detail. Nevertheless, the limited evidence available suggests that Shh signals in the spinal cord drive morphine-induced hyperalgesia (MIH) and tolerance primarily through the upregulation of BDNF, thereby suggesting that Shh may be a mediator of MIH regulation and tolerance. Moreover, the inhibition of Shh signalling, mainly during early phase development, delays or sometimes completely suppresses MIH and its tolerance [76]. The Shh signalling pathway can modulate BDNF expression in normal and regenerating cavernous nerves (CN), which are frequently injured during prostatectomy. The manipulation of the nerve microenvironment is extremely critical to hasten the regeneration of CN post-trauma, and therefore should be studied further to identify novel therapies for erectile dysfunction. Shh treatment can improve CN regeneration in association with BDNF, which may drive the return of erectile function after CN trauma, suggesting that BDNF may be a target of Shh not only in CN but also in cortical neurons and the sciatic nerve [77]. Further, the connection between Notch and BDNF expression is unexplored and should be investigated.

## 7. Developmental signalling and its role in modulating mitochondrial dynamics

**Figure 3.**
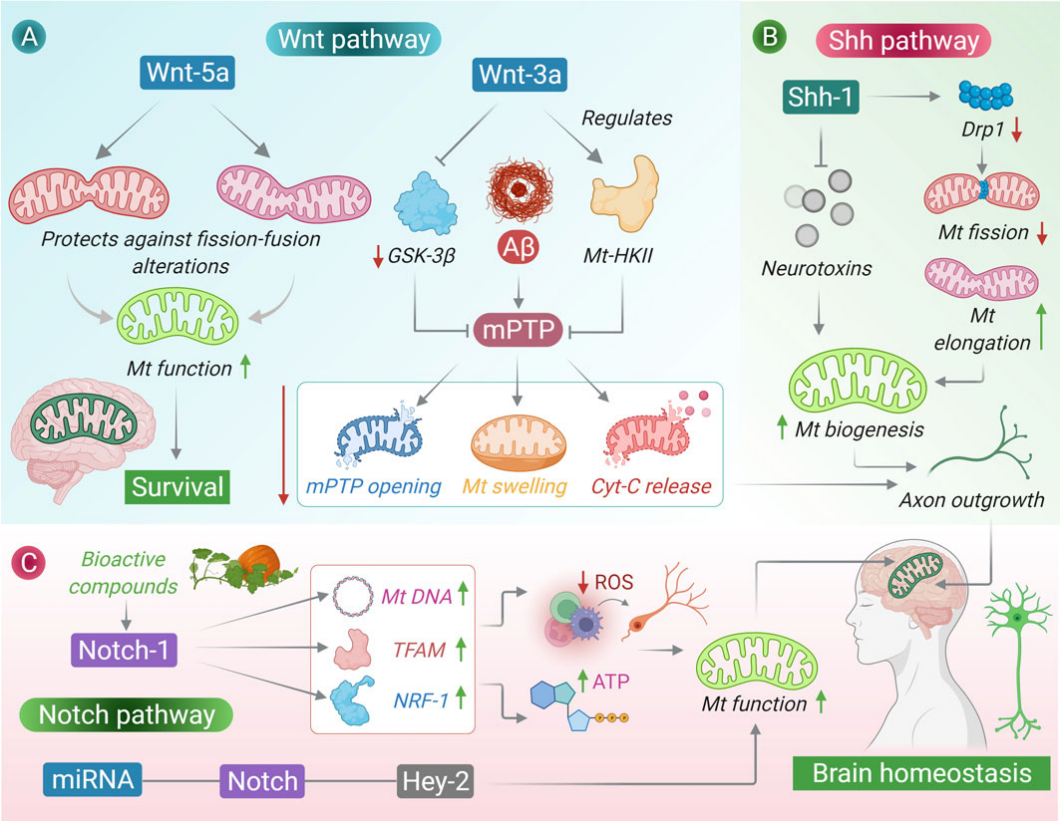
Interplay between mitochondrial biogenesis and developmental signalling pathways in the CNS. (A) Wnt signalling pathway and mitochondrial dynamics, (B) Hedgehog signalling pathway and mitochondrial dynamics, and (C) Notch signalling pathway and mitochondrial dynamics.

Mitochondria are the lifeline of all eukaryotic cells. In addition to their primary role in generating cellular energy in the form of adenosine triphosphate (ATP), mitochondria are highly involved in several other cellular processes such as calcium buffering and apoptosis. In neurons specifically, mitochondria are involved in the development of nascent neurons and in defining the synaptic plasticity of mature neurons [78,79]. Aberration of mitochondrial functions in neurons has been linked to the pathogenesis and prognosis of neurological disorders such as ischaemic stroke and AD [80]. Several studies have suggested that the mitochondrial functions are subtly controlled and driven by an array of growth factors and signalling cascades such as the developmental pathways (figure 3). In hippocampal neurons, the activation of the Shh signalling cascade has been shown to affect key aspects of mitochondrial dynamics such as the mitochondrial mass, which is significantly greater in neurons treated with the Shh stimulus. The enhancement of mitochondrial functions by the Shh signalling pathway is critical towards Shh-stimulated axon outgrowth [81]. Further, some articles suggest that Shh-activity limits mitochondrial fission and augments mitochondrial elongation, primarily by suppressing the expression of mitochondrial fission protein dynamin-like GTPase, Drp1. Moreover, compared with mitochondria in Shh-untreated neurons, those in Shh-treated neurons are more electron-dense with higher membrane potential and respiratory activity, indicating the key involvement of the Shh signalling pathway in driving mitochondrial dynamics in the CNS. In addition, studies have reported that Shh signalling offers some type of neuroprotection to the neurons against the mitochondrial poison rotenone, Aβ-peptide, hydrogen peroxide and high levels of glutamate; thus, further strengthening the hypothesis [47]. Although few studies have studied this topic, the available data emphasize a strong connection between the Shh pathway and the physiological properties of mitochondria in the CNS [82].

Notch-mediated mitochondrial biogenesis and functional improvement have been recently studied in the oxygen-glucose deprivation (OGD) model of ischaemic injury. It was found that the treatment of OGD cortical neurons with rosuvastatin (RSV) restricted the generation of ROS and significantly upregulated the mitochondrial activity (as measured through the ATP levels) in damaged cortical neurons. Further, RSV treatment augmented the mitochondrial DNA content and enhanced the mRNA and protein level of mitochondrial transcription factor A and nuclear respiratory factor 1. However, silencing of Notch1 activity in primary cortical neurons completely reversed RSV-induced mitochondrial biogenesis under OGD conditions. Nonetheless, these observations suggest that RSV can restore neurite outgrowth in cortical neurons damaged by OGD *in vitro*, partly by improving mitochondrial biogenesis through a Notch-1-mediated mechanism [83]. In other neurodegenerative conditions such as AD, the Notch downstream effector Hey-2 has been shown to interact with miR-98, reduce Aβ production and oxidative stress, and improve the mitochondrial function in AD models [84]. Finally, the Wnt signalling pathway has a strong connection with mitochondrial dynamics, especially within the CNS. For instance, Wnt-3a-mediated activation of the canonical Wnt signalling pathway limits the permeabilization across the mitochondrial membranes by limiting the transport across the mitochondrial permeability transition pore (mPTP) induced by toxic Aβ oligomers in an AD mouse model [85]. Further, Wnt-5a-mediated activation of the non-canonical Wnt signalling pathway confers some type of protection to the mitochondria against fission–fusion alterations in AD. Wnt signalling regulates mPTP permeability possibly through the following two mechanisms: first, by downregulating the expression of mitochondrial GSK-3β, and second, by regulating the mitochondrial hexokinase II mRNA and protein expression levels and activity. Overall, these findings suggest that the Wnt signalling pathway regulates the Aβos-induced cascade of mitochondrial events such as mPTP opening, mitochondrial swelling, mitochondrial membrane potential loss and cytochrome c release. Hence, any deregulation in the Wnt signalling pathway can have detrimental effects such as neuronal death [86]. These data emphasize the fact that the engagement of developmental signals in memory development and memory loss through the modulation of mitochondrial dynamics is extremely crucial for maintaining normal neuron and brain homeostasis, and hence should be explored in detail to identify disease–critical interactions.

## 8. Developmental pathways and neuroinflammatory responses in the central nervous system

Neuroinflammation is a typical immune activity that is triggered in response to any type of brain trauma, injury and disorder. It helps in protecting the CNS from severe damages and in restoring normal brain homeostasis post-trauma. In addition to modulating diverse cellular processes, developmental pathways drive a panel of neuroinflammatory activities in the brain. The Wnt signalling pathway plays a major role in modulating the post-trauma neuroinflammatory processes in the CNS [87–89]. Several studies have reported that the canonical Wnt signalling pathway has anti-inflammatory effects, whereas the non-canonical Wnt signalling pathway has pro-inflammatory effects [87,88]. The anti-inflammatory processes driven by the activation of the canonical Wnt pathway are the result of its direct interaction with the key members of the NF-κB transcription factor family, such as RelA [87,90,91]. The non-canonical pathway also interacts and induces the NF-κB transcription factor. In addition, there is evidence regarding the involvement of several other signalling components such as PI3 K/AKT, Ras-related C3 botulinum toxin substrate 1 (Rac1) and mitogen-activated protein kinase (MAPK) in the process [92]. Moreover, the activation of the non-canonical Wnt pathway in response to Aβ exposure helps build a pro-inflammatory environment within the microglia and amplifies the neuroinflammatory state [88,91,93]. Evidence shows that despite being pro-inflammatory, the non-canonical Wnt-5a ligands may sometimes exert anti-inflammatory effects, especially in bone tissue challenged with lipopolysaccharide (LPS). Similarly, canonical Wnt ligands can trigger a pro-inflammatory state, especially within previously activated microglia [94–96]. Overall, these contradictory findings point towards the fact that the physiological and cellular context of the Wnt signalling pathway is extremely crucial when deciphering the role of the Wnt signalling pathway during neuroinflammation.

The Wnt and Toll-like receptor (TLR) signalling pathways together can downregulate the canonical Wnt signals and help to drive the neuroinflammatory processes in the CNS [87]. TLR4 activation prevents low-density lipoprotein receptor-related protein 6 (LRP6) phosphorylation required for Fz-LRP5/6 activity that results in the inhibition of the canonical Wnt signalling at a very early stage of the pathway [97]. In macrophages, the activation of the non-canonical Wnt signalling pathway (Wnt/Ca^2+^) by the ligand Wnt-5a triggers the expression of the suppressor of cytokine signalling 1 and protein inhibitors of activated STAT-1 in a TGF-β activated kinase-1 (TAK1)-dependent manner. This ultimately results in a decrease in the expression matrix of a panel of signal transducers of the TLRs cascade, such as IRAK members and MyD88 [98]. Further, GSK-3β, the key member of the Wnt signalling pathway, is extremely critical towards TLR-mediated cytokine production, and the silencing of GSK-3β impairs the ability of NF-κB to bind to the CREB-binding protein [87,90,99]. This impairment may be a result of Wnt hyperactivation and possible nuclear β-catenin localization, which is enhanced in response to some type of trauma or disorder. Further evidence on the involvement of GSK-3β node was provided by Li *et al.* [100] while studying the effects of lithium, a well-known canonical Wnt signalling agonist that inhibits GSK-3β activity. The study further reported that lithium not only diminishes the expression of pro-inflammatory effectors such as interleukin (IL)-6 but also reduces the expression of TLR4, especially in astrocytes [100]. Finally, toxic substances such as fluoride can drive neuroinflammation in the CNS by altering the Wnt signalling pathway in some form in BV2 microglial cells. This provides a strong basis for the fluorine-induced neuroinflammation injury theory [101]. A convergence around the idea that GSK-3β supports the crosstalk between the TLR, NF-κB and Wnt signalling pathways exists, irrespective of whether these effects are regulated directly or indirectly through the mediators of the Wnt signalling pathway. These interactions may altogether be part of a higher-order immune or inflammatory response driven by the Wnt signalling pathway in the CNS.

In the CNS, the Notch signalling pathway is central to processes known to regulate the normal development of NPCs, neurons, oligodendrocytes and astrocytes [102–106]. However, recent evidence suggests that Notch may be involved in driving the immune cell response against any type of stimulation, infection, trauma or disorder [107–110]. In addition, Notch-1 is upregulated in the postnatal rat brain microglia when challenged with the bacterial toxin LPS. Moreover, the inhibition of Notch-1 concomitantly downregulates the mRNA and protein expression of IL-6, IL-1 and inducible nitric oxide synthase and upregulates the mRNA expression of TNF-α in the microglia [111]. In the CNS, microglial cells are one of the most important immune cells that perform diverse roles, especially during neuroinflammatory diseases. The behaviour of Notch1 in microglial cells suggests that the signalling plays a putative role in regulating microglial maturation and activation under neuroinflammatory stress [110–113]. In hypoxic brain injury, the canonical Notch signalling pathway drives microglia activation, which can be linked to multiple immuno-pathological events in the brain. The expression levels of Notch-1 and Delta-1 are reportedly higher, and a significant thrust in the expression of NICD, RBP-J*κ* and Hes-1 is observed in primary microglia and BV-2 microglial cells in the post-hypoxic stress condition compared with the normal condition. Chemical inhibition of the Notch signalling pathway with GSIs such as DAPT reverses the hypoxia-mediated effects of a range of the Notch mediators. Furthermore, GSI-mediated Notch inhibition limits the expression and translocation of NF-κB/p65 and suppresses the activation of the TLR4/MyD88/TRAF6 pathway. These observations emphasize the close interrelationship between the Notch and NF-κB signalling pathways and the strong possibility of the TLR4/MyD88/TRAF6/NF-κB signalling axis being operational during neuroinflammation [114].

The Notch signalling pathway can steer an immune response through the expression of several cytokines and effector proteins by immune cells like macrophages. In haematopoietic progenitor cells, the Notch signalling pathway is activated by a range of pro-inflammatory stimuli such as TNF-α and LPS [115–118]. Recent studies have suggested that Notch and NF-κB signalling pathways together regulate the response to injury and cytokines in the CNS, where NF-κB stabilizes the hypoxia-inducible factor (HIF)1-CSL-NICD complex to facilitate the transcription of bHLH genes such as Hes. NF-κB can modulate and integrate with the Notch signalling pathway through both extrinsic (through Notch ligands) and intrinsic (through intracellular Notch modulators) mechanisms [119]. A study reported that pre-treatment with GSIs or silencing of Notch-1 prevents the translocation of NF-κB p50 into the nucleus upon LPS/IFN-γ induction [117,119]. Therefore, complex interactions between the Notch and NF-κB signalling pathways may be involved in attenuating or augmenting key inflammatory responses in preterm brain post-intrauterine infection or a neuroinflammatory event.

Insufficient data are available to comprehensively evaluate the role of the Shh signalling pathway during neuroinflammation. However, one study deciphered the potential interaction between a major pro-inflammatory cytokine (IL-1β) and the Shh signalling pathway. IL-1β is released from activated microglia. It increases the permeability of BBB. IL-1β reduces the protective effect of astrocytes on BBB integrity, primarily by suppressing the astrocytic Shh activation. Astrocyte conditioned media, Shh or Shh signal agonists reverse the effect and strengthen the BBB integrity by upregulating the TJ proteins, whereas Shh signal inhibitors completely abrogate these effects. Moreover, IL-1β and the Shh signalling pathway promote astrocytic production of pro-inflammatory chemokines such as monocyte chemoattractant protein-1, MCP-1 (CCL2), chemokine (C-C motif) ligand 20 (CCL20) and C-X-C motif chemokine ligand 2 (CXCL2), which induce immune cell migration and exacerbate BBB disruption and neuroinflammation [40]. Although these observations are engaging, further research is required to understand mechanisms by which the Shh signalling pathway drives neuroinflammatory processes in the CNS.

## 9. Alzheimer's disease: a grand alliance of developmental signalling pathways

**Figure 4.**
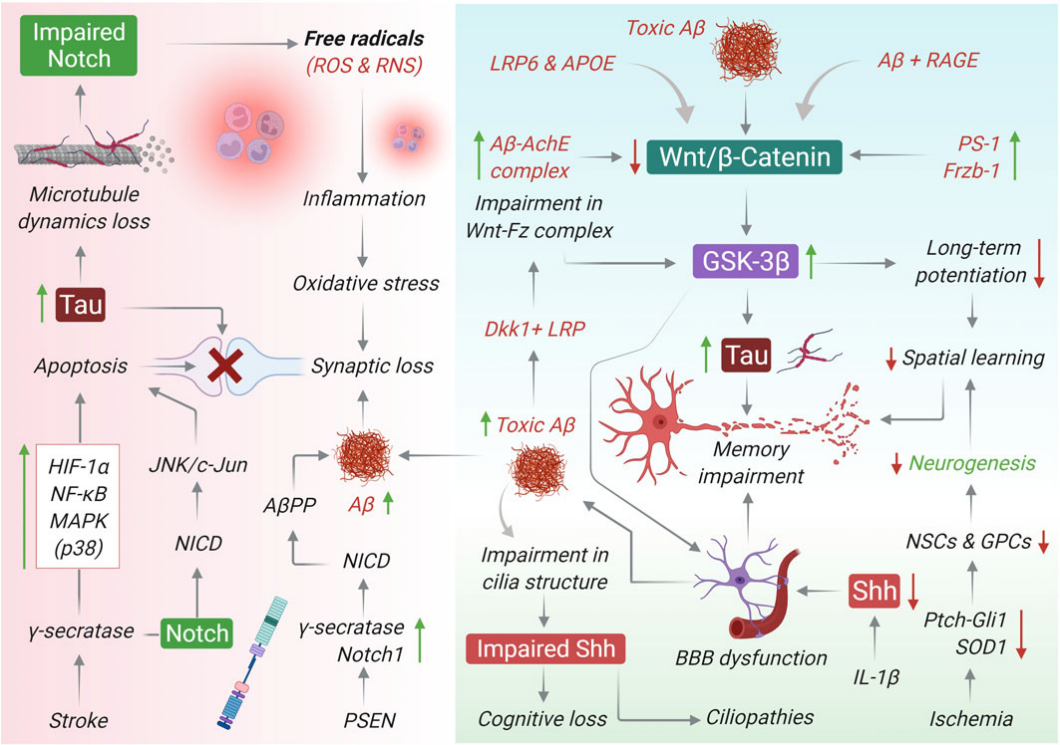
Developmental signalling pathway crosstalk during brain impairment. Numerous studies have shown an aberration in the Notch signalling pathway and its associated components in the case of AD. Mutations in the genes encoding for AβPP, PS-1 and PS-2 have been held accountable for early onset of the disease. Besides, in post-mitotic neurons, in addition to PSs, Notch has also been found to interact with AβPP while driving memory deficits linked with AD. There is a potential association between apoptosis and Notch signalling following stroke and AD. Notch signalling may contribute to the pathogenesis of oxidative stress in cerebrovascular diseases and synaptic loss. Additionally, impaired Notch can lead to microtubule dysfunction and Tau phosphorylation. Further, reports suggest that Aβ peptides can disturb the normal activation and operation of the Wnt/β-catenin pathway in the AD brain. Among all the Wnt-associated components that are affected, β-catenin has emerged as the one repeatedly downregulated in neurons displaying PS-1-inherited mutations. Toxic Aβ causes downregulation of Wnt, which leads to an increase in GSK-3β and Tau phosphorylation and subsequently memory loss. In addition, it also contributes to an increase in GSK-3β and Tau levels following impairment in the Wnt-Fz complex induced by Dkk1 and LRP complex. Dkk1 can also reversibly lower the number of synaptic proteins and the number of active pre-synaptic sites, thereby triggering synaptic disassembly at pre- and post-synaptic neuron sites. Apolipoprotein E (apoEε4), a potential risk factor in AD, also blocks canonical Wnt activation. Higher expression of Aβ-AchE complex and Frzb-1 can also downregulate Wnt activation. Further, dysfunctional Wnt/β-catenin signalling causes BBB breakdown in AD. Toxic Aβ can impair cilia structure and associated impairment in Shh signaling, leading to cognitive loss and ciliopathies. Similarly, IL-1β abolishes the protective effect of astrocytes on BBB integrity by suppressing astrocytic Shh production and ultimately leads to CNS dysfunction. Lastly, ischaemia can also decrease the neurogenesis process via downregulation of Ptch, Gli and SOD1 components of Shh signalling.

AD is an age-associated neurodegenerative disorder (NDD) characterized by the progressive loss of cognitive function and memory, and other associated neurobehavioural problems [120,121]. AD has two common biological markers: first, neurofibrillary tangles in the intracellular space of neurons. These structures are a result of the sequential aggregation of the tau protein after hyperphosphorylation, a process that progresses through the assembly of oligomeric structures called paired helical filaments (PHFs) [122–126]. Second, senile plaques are formed by the deposition of Aβ peptides (39–42 amino acid residues) in the extracellular environment. These peptides are derived from the amyloid precursor protein (AβPP) as a result of proteolytic excision by the enzymes β and γ secretases. These two markers promote loss of synaptic processes and, finally, neuronal death [127,128]. Furthermore, there is evidence that the onset of AD is in part due to ‘damage signals' or tau oligomers generated through microglial cells. These signals trigger a neuroinflammatory response, promoting the misfolding of the cytoskeletal structure and generating a positive feedback loop that subsequentially promotes neuronal damage [124,129,130]. Mutations in AβPP, presenilin-1 (PS-1) and presenilin-2 (PS-2) genes are well-known drivers of disease prognosis in AD. Moreover, emerging studies suggest that there exists a strong interplay between the embryonic signalling pathways and other associated components that define the pathological outcomes of memory impairment in patients with AD (figure 4) [131,132]. In this section, we explore the major interactions that might be fundamental from a therapeutic viewpoint in AD.

### 9.1. Notch-based interactions in Alzheimer's disease

Notch, the key substrate of c-secretase/presenilin, plays a role in learning and memory-related processes, thereby providing a foundation to explore the potential link between the Notch signalling pathway and the pathogenesis of AD. Mutation in genes encoding AβPP, PS-1 and PS-2 is associated with the early onset of AD. In addition to PSs, Notch has been found to interact with AβPP while driving memory deficits linked with AD in post-mitotic neurons. Moreover, loss-of-function of the Notch gene has been reported to result in neuronal dysfunction and long-term spatial memory deficits, as observed in the Notch mutant mouse model having normal acquisition and short-term spatial memory [133]. A chronic decrease in Notch signalling was found to drive specific learning and memory deficits, which indicates that Notch-dependent transcriptional regulation is critical for spatial learning and memory. Interestingly, Notch expression has been found to be elevated up to two folds in the brains of patients with AD compared with age-matched control subjects. Moreover, the Notch signalling pathway and its associated components such as Delta-like 1 (Dll1) and Hes-1 have been reported to be hyperactivated in the cortex of patients with Down syndrome, as observed in AD. However, Down syndrome fibroblasts and AD cortex show similar overexpression patterns with respect to Notch-1 and Dll1, thereby suggesting that augmented Aβ production and neurodegeneration [134]. Although the prognostic mechanisms of NDDs vary greatly, it is now becoming increasingly clear that the Notch signalling pathway may be involved to a large extent in the neuronal dysfunction observed in most of these diseases [133]. However, a lot remains to be studied for the molecular mechanisms of perturbations of the Notch signalling pathway in the AD brain to answer whether aberrant Notch signalling in AD can be pathophysiologically significant for neurotherapeutic intervention. Although downregulation of Notch1 expression affects synaptic plasticity, memory and olfaction, its upregulation after brain trauma can be extremely harmful to neuronal survival. Studies have suggested that familial AD mutations in the PS affect Notch-1 expression and processing; however, some studies have suggested that Notch-1 may be overexpressed in sporadic AD because of similar mutational events. For instance, immunohistochemical analysis of Notch-1 in post-mortem cortical and hippocampal samples revealed an accumulation of Notch-1 in plaque-like structures in the brain parenchyma of patients with sporadic AD. In many cases of AD, Notch-1 has been found to be associated with fibrillary tangles or plaques. Nevertheless, it is essential to understand that Notch deregulation may be considered a novel hallmark of AD prognosis [133,135].

### 9.2. Shh-based interactions in Alzheimer's disease

Studies have suggested that Aβ peptides can disturb the normal activation and operation of the canonical Shh signalling pathway in the AD brain [136]. Self-aggregation of the Aβ peptide is a characteristic feature in the hippocampus of patients with AD and the Shh signalling pathway may be involved in the process. Cell culture and animal studies have indicated that the Shh signalling pathway impairs hippocampal neurogenesis and synaptic plasticity that in turn triggers excitotoxic neuronal Ca^2+^ overload [137–141]. Recently, studies have started extensively investigating the possible involvement of Shh in the pathogenesis of AD. Brain tissue samples extracted from patients with AD and AβPP mutant mice have extremely low levels of Ptch and Gli-1, suggesting subtle deregulation of the Shh signalling pathway and its components [142]. Further, the ability of Shh to mediate nerve growth factor (NGF) effects on cultured basal forebrain cholinergic neurons supports the role of the Shh signalling pathway in AD progression. These effects not only depend on an increase in the proliferation of cholinergic precursors but also post-mitotic phenomenons such as enhanced survival of differentiated neurons or recruitment of neuronal cells to the cholinergic lineage from a post-mitotic pool [143]. Reduction of neurogenesis in the brain is one of the chief drivers of dementia in AD; on the contrary, modifying and/or improving the course of hippocampal neurogenesis has beneficial effects in patients with AD symptoms. Although the expression levels of Ptch-1 and Gli-1 are substantially higher at early ages, a significant decrease in the Ptch-1 and Gli-1 levels have been observed in the hippocampus of aged AD transgenic mice; this can compromise the ability of genesis in both NSCs and glial precursor cells. Overall, these observations suggest that the deregulation of the Ptch-1–Gli-1/Shh signalling pathway may result in detrimental loss of NSCs and glial precursor cells, thereby contributing to serious cognitive decline in AD brains [142,144].

### 9.3. Wnt-based interactions in Alzheimer's disease

Similar to the Notch and Shh signalling pathways, the Wnt pathway has been attributed to play a role in AD biology. Numerous studies have reported an aberration in the Wnt signalling pathway and its associated components in AD. Among all the Wnt-associated components that are affected in AD, β-catenin is repeatedly downregulated in neurons displaying PS-1-inherited mutations [145]. PS-1 regulates GSK-3 activity and tau microfibrillar phosphorylation through a Wnt-dependant mechanism and PS-1 mutations have been shown to enhance both GSK-3 activity and tau phosphorylation in the absence of Wnt signals [146–148]. As mentioned above, tau hyperphosphorylation allows the formation of oligomers and thereafter, PHFs and neurofilament light chain (NFL), the ‘damage signals', trigger a neuroinflammatory response by activating microglial cells. These events promote an abnormal signalling cascade, involving the release of NF-κB, increase in cyto- and chemokines levels and subsequent activation of receptors in the neuron, triggering an overexpression of the cyclin-dependent kinase 5 (CDK5)/p35 complex, GSK-3β and tau hyperphosphorylation. The new hyperphosphorylated tau self-aggregates and results in neuronal cell damage. Several studies have suggested that after neuronal apoptosis, the release of tau oligomers and polymers promote the reactivation of microglial cells, generating a positive feedback loop of this altered molecular signalling responsible for neuron degeneration in tauopathies and AD [123]. Furthermore, exposure of cultured hippocampal neurons to toxic Aβ peptides results in the inhibition of the canonical Wnt signalling pathway, and subsequent accumulation of Aβ leads to the activation of Dickkoff-1 (Dkk1), a potent Wnt protein antagonist [149–151]. In line with these observations, increased Dkk1 levels have been reported in post-mortem brain samples derived from patients with AD and transgenic AD animal models [152,153]. In addition, Dkk1 can reversibly lower the number of synaptic proteins and the number of active pre-synaptic sites, thereby triggering synaptic disassembly at pre- and postsynaptic neuron sites [154,155]. In addition, clustering, a susceptibility factor that is known to drive the late onset of AD, regulates Aβ toxicity through Dkk1-driven initiation of the non-canonical Wnt/PCP–JNK signalling pathway, resulting in tau phosphorylation and cognitive impairment [156]. Another protein called Dkk3, which is closely related to Dkk1, has been reported to be upregulated in the plasma and cerebrospinal fluid (CSF) of patients with AD [157]. Overall, these observations suggest a potential role of the Dkk protein in regulating the Wnt activity in the AD brain. Many more critical Wnt-based interactions are being deciphered, which demands further investigation. For instance, apolipoprotein E (apoEε4), a potential risk factor of AD, blocks the activation of the canonical Wnt signalling pathway. It was reported that genetic variations within the LRP6 enhance the apolipoprotein E-mediated inhibition of the Wnt signalling pathway during AD progression [158–160]. A recent study by Tapia-Rojas *et al.* [161] found that Wnt signalling promotes AβPP processing by controlling the expression of the enzyme β-secretase (BACE1) through several downstream effector molecules. Moreover, the siRNA-mediated inhibition of BACE1 decreases the Aβ accumulation, as observed in plasma and CSF samples from AD brains [161,162]. Therefore, it can be said that Wnt/β-catenin signalling inhibition increases amyloidogenic AβPP processing, that is, Aβ peptide (1–42) formation and their aggregation in AD brains. Hence, amyloid deposition in the AD brain is because of a compromised BBB, and therefore there exists an imbalance between Aβ deposition and its clearance. Microglial cells play a fundamental role in maintaining normal brain homeostasis and in Aβ clearance through several phagocytic and digestive processes. There is strong evidence that the regulation of phagocytosis and survival of microglia during Aβ clearance may be controlled in part by an innate immune receptor called triggering receptor expressed on myeloid cells 2 (TREM2) and TREM2 activates the Wnt/β-catenin signalling pathway, thereby suggesting strong participation of the Wnt/β-catenin signalling pathway in Aβ clearance [163].

Finally, emerging reports have highlighted the possible role of Wnt deregulation in the aetiology of both types of AD. A dysfunctional Wnt/β-catenin signalling pathway causes BBB breakdown in AD. However, in-depth studies are warranted to establish such Wnt-associated mechanisms, which significantly define the neurodegenerative properties of AD [164]. For instance, a methionine-enriched diet can trigger memory impairment complemented by the loss-of-function of the Wnt signalling pathway. Methionine decreases the level of active β-catenin in neurons, resulting in a substantial increase in the activity of GSK-3β along with a reduction in the expression levels of the Wnt target genes such as cyclin D1 and c-Jun. These results essentially indicate a hiatus in the activity of the entire Wnt signalling pathway. In addition, these results suggest that similar to several other proteins and factors, l-methionine can induce a loss of Wnt signalling, as observed in AD [165].

## 10. The role of developmental signalling pathways in other neurological abnormalities and disorders

### 10.1. Parkinson's disease

PD is the second most prevalent NDD and is currently incurable. PD is characterized by the progressive loss of a subset of midbrain dopaminergic (DA) neurons in the substantia nigra region, which leads to the loss of motor ability [166]. Disease-causing biology of PD is poorly understood and can be associated in part with the rising number of genetic defects that define an array of pathological outcomes in PD. Parkin (PARK) and Wnt interactions are extremely critical during PD pathogenesis, and hence any aberration in the Wnt signalling pathway can disturb the expression levels of PARK in neurons, driving PD progression [167,168]. Further, proteins encoded by PARK genes have been shown to alter the Wnt signalling pathway in some form. Therefore, any subtle aberration in the expression PARK genes and/or associated impairment in the Wnt signalling mutational events can contribute towards PD progression [128]. In a rotenone-induced *Drosophila* model of PD, impaired Wnt signalling was found in dopamine-containing neurons and was found to be associated with PD pathogenesis. Further, transcriptome analysis revealed that the genes associated with regulation of cell death and neuronal functions were significantly upregulated along with pathways such as the MAPK/EGFR- and TGF-β signalling pathways. The Wnt signalling pathway was found to be significantly downregulated; however, upregulation of the Wnt signalling pathway by ectopic overexpression of armadillo/β-catenin led to a complete reversal of the rotenone-induced movement impairments in the PD model [169]. Another biologically important protein, the leucine-rich repeat kinase 2 (LRRK2), has been found to be associated with the Wnt signalling pathway, especially in autosomal dominant familial and sporadic PD. LRRK2 is a large GTPase and kinase domain-containing protein; mutations in LRRK2 have been detected in a large proportion of PD cases in certain populations. MAPK1 and MAPK3 (also known as ERK1/2) and Wnt could be potential downstream mediators of mutant LRRK2 effects. Strong evidence suggests that the effect of LRRK2 mutations is diverse and includes microtubule dynamics, protein synthesis, autophagy and signalling crosstalk involving the ERK1/2 and Wnt signalling cascades [170,171]. Finally, epigenetic mechanisms play a key role in deregulating the Wnt activity and the same has been observed during PD progression. In a study by Zhang *et al.* [172], genes from the Wnt signalling pathway responsible for neurogenesis were hypermethylated in PD brains compared with their matched controls. Moreover, consistent with these DNA methylation changes, a significant reduction in the mRNA and protein levels were observed for four Wnt and neurogenesis-related genes, namely Forkhead box C1 (FOXC1), Neurogenin 2 (NEURG2), Sprouty RTK signalling antagonist 1 (SPRY1) and catenin beta 1 (CTNNB1), in the midbrain dopaminergic neurons of PD brains. Further, treatment of DA neurons with a low concentration of 1-methyl-4-phenylpyridinium (MPP^+^) results in the downregulation of Wnt-associated factors. Overall, this study revealed a key link between epigenetic mechanisms and Wnt signalling and its connection to pathogenesis and progression of PD [172].

LRRK2 protein not only interacts with Wnt but also activates the Notch signalling pathway, primarily through the endosomal pathway. Studies have suggested that two novel LRRK2-associated proteins, a HECT-type ubiquitin ligase (HERC2) and an adaptor-like protein with neuralized domains (NEURL4) may be principally involved in the modulation of the Notch signalling pathway. LRRK2 can bind to NEURL4 and HERC2 through the LRRK2 Ras of complex proteins (ROCs) domain and NEURL4 domain, respectively. It has been suggested that HERC2 and NEURL4 potentially link LRRK2 to the cellular vesicle transport pathway and Notch signalling pathway, through which the LRRK2 complex facilitates the recycling of the Notch ligand Dll1 by modulating the endosomal trafficking pathway. This mechanism negatively modulates the Notch signalling pathway mainly through *cis*-inhibition by stabilizing Dll1, which in turn augments NSCs differentiation and regulates the functioning and survival of differentiated DA neurons. LRRK2 mutations such as the R1441G ROC domain-mutations intensify the activation of the Notch signalling pathway through the endosomal pathway, thereby emphasizing that the deregulation of Notch activity in mature neurons is a characteristic of PD aetiology and is linked to *LRRK2* [173].

Insufficient data are available to establish the role of the Shh signalling pathway in PD. Nevertheless, the disruption of a non-cellular autonomous mode of Shh signalling originating from DA neurons has been shown to drive progressive, adult-onset degeneration of dopaminergic, cholinergic and GABAergic neurons in the mesostriatal circuit. In addition, the imbalance of cholinergic and dopaminergic neurotransmission and motor deficits are observed in PD. Further, variable Shh signalling can result in stepwise inhibition of muscarinic auto-receptor and glial cell line-derived neurotrophic factor (GDNF) expression mainly in the striatum. In addition, graded signals that originate from striatal cholinergic neurons and interact with the canonical GDNF receptor (Ret) can completely block Shh expression and activity in DA neurons. These results provide integrative insights into non-cell-autonomous processes involving the Shh signalling pathway, which is likely during neurodegenerative conditions such as PD [174].

### 10.2. Amyotrophic lateral sclerosis

ALS is a neurodegenerative trauma characterized by the death of upper and lower motor neurons (MNs) [175]. Although the cause of death of MNs is not clearly understood, some studies have confirmed that the defective embryonic signalling pathways directly or indirectly participate in ALS pathogenesis. To begin with, the Wnt/β-catenin signalling pathway has been shown to modulate the degeneration of MNs, and therefore, it is actively researched using *in vitro* ALS models. Several studies have reported that the activity of the canonical Wnt/β-catenin pathway and its associated members such as Wnt-2, Wnt-3a, Wnt-7a, Wnt-5a, Fzd1, Fzd2, β-catenin, Cyclin D1 and GSK-3β is largely deregulated in the astrocytes and MNs extracted from the spinal cord of G93A superoxide dismutase-1 (SOD1) transgenic mouse model of ALS [176–179]. Both Wnt2 and Wnt7a mRNA and protein expression have been found to be upregulated in the spinal cord of ALS mice compared with wild type. Moreover, the immune-reactivity of Wnt-2 and Wnt-7a has been found to be strong in an adult transgenic mouse model of ALS and weak in wild-type mice. The degeneration of MNs results in an upregulation of the expression of Wnt-2 and Wnt-7a in the spinal cord of ALS mice, which in turn augments Wnt activity and blocks GSK-3β activity as seen in an adult transgenic mouse model of ALS [177–179]. In particular, the aberrant cellular distribution of Fz5 can be considered a good prognostic marker for ALS progression, which might be indicative of a pathophysiological role of the Wnt signalling pathway in neurons with increased levels of Wnt and/or Fzd expression [180]. Finally, cytosolic accumulation of β-catenin has been reported in an *in vitro* model of ALS with a G93A mutated form of human Cu/Zn SOD1. Further, β-catenin has been found to be activated in myofibres in extraocular muscles and limb muscles in patients with ALS. These observations collectively suggest that the Wnt/β-catenin signalling axis plays a fundamental role in the neurodegeneration of MNs in ALS [181,182].

Studies have indicated that the Shh signalling pathway may be compromised in patients with ALS. To ratify the hypothesis, a group of researchers measured the protein and biological activity levels of Shh in the fluid surrounding the brain and spinal cord (CSF) of patients with ALS and healthy subjects (people without ALS). Additionally, they tested whether these levels correlated with the severity and progression of ALS. In total, they measured the Shh level in 9 patients with ALS, 12 patients with another neurological condition and 13 healthy subjects who were undergoing spinal anaesthesia for hip or knee replacement. Initially, they found no difference in the expression levels of the Shh protein between patients with ALS and healthy subjects. However, in the CSF from patients with ALS and control subjects (after induction of Shh activity), the activity of Shh in the CSF was increased in healthy control subjects (as expected), but not in patients with ALS. The study concluded that the CSF of patients with ALS should contain an inhibitor that blocks Shh signalling. Nevertheless, the inhibitory effect in the CSF of patients with ALS might not correlate with ALS disease severity. However, the increasing levels of cell signalling proteins, IL-1β and TNF-α, may correlate. In line with this, IL-1β and TNF-α levels were found to be elevated in the CSF of patients with ALS and the higher the levels, the quicker the disease progressed. Remarkably, TNF-α substantially inhibited the Shh activity *in vitro*, thereby suggesting a novel role of TNF-α and Shh in the development and progression of ALS [183].

Finally, the deregulation of the Notch signalling pathway has been seen as a key driving factor behind reduced neurogenic response in the hippocampus of patients with ALS [184]. Further, such deregulation has been observed in the spinal cord of SOD1G93A mice and *in the spinal cord of infirm* with sporadic ALS (sALS). The increased activation of Notch can be seen within the population of reactive GFAP-positive astrocytes. In fact, one of the key Notch ligands (Jagged-1) was found to be ectopically expressed in reactive astrocytes in the spinal cord from mice and patients with ALS but was unexpressed in resting astrocytes. The astrocyte-limited inactivation of Jagged-1 in presymptomatic SOD1G93A mice can exacerbate the activation of the Notch signalling pathway and worsen the course of the disease in these models without affecting disease onset. This suggests that aberrant activation of the Notch signalling pathway contributes significantly to the pathogenesis of ALS, both in patients with sALS and SOD1G93A mice, and is driven in part by the upregulation of astrocytic Jagged-1 [185].

### 10.3. Diabetic neuropathy

Several studies are now investigating the involvement of embryonic signalling pathways in diabetic neuropathy, one of the long-term complications of diabetes [186]. For instance, Wnt signalling affects a range of cell types, namely embryonic stem cells, neural cells and mammary cells, and deregulated Wnt-1 activity has been reported in patients with diabetes [186,187]. GSK-3 is a well-known participant in the Wnt signalling pathway. Studies have indicated a connection between GSK-3 and an increase in insulin receptor phosphorylation in patients with diabetic neuropathy and experimental models of diabetic neuropathy [188–195]. Therefore, GSK-3 can be explored as a promising target in many complex diseases such as peripheral diabetic neuropathy, and for neural protection and neuropathic pain reduction [192,194,196]. Another possible link can be established between hypertension, the insulin signalling pathway and the canonical Wnt signalling pathway in diabetic nephropathy. This has been validated in a study that reported that the downregulation of the canonical Wnt signalling pathway in the nucleus tractus solitarii results in an increase in phosphorylation of insulin signalling proteins [197]. One of the key characteristics of diabetic nephropathy is excessive deposition of extracellular matrix proteins in the mesangium, tubulointerstitium of the glomerulus and basement membrane, leading to mesangial expansion and renal fibrosis [198]. The Wnt/β-catenin signalling pathway plays a key role in the progression of diabetic neuropathy; however, downregulation of the Wnt/β-catenin pathway can have adverse effects on kidneys, such as increased apoptosis of mesangial cells, enhanced deposition of fibrous tissue in the mesangium, epithelial-mesenchymal transition (EMT), podocyte and renal injury and fibrosis [199–204]. Overall, these observations indicate the importance of the Wnt signalling pathway in determining the pathophysiology of diabetes and support the possibility of exploring the role of Wnt signalling as a potential therapeutic target.

Similarly, impaired Shh signalling-mediated endothelial dysfunction (microangiopathy) may be a key factor in driving diabetic neuropathy. Studies have suggested that downregulated Dhh expression, as seen in diabetic nerve, can at least partly contribute to the development of neuropathy through its action on vasa nervorum, where Dhh is critically involved in maintaining blood–nerve barrier integrity. This highlights for the first time that endothelial dysfunction driven by altered Dhh expression may be sufficient to induce and drive neuropathy [205]. Further, the Notch signalling pathway has been studied in diabetic retinopathy, and crosstalk between the Notch-1 and TLR4 signalling pathways has been reported to be one of the key mechanisms in the development and/or progression of diabetic neuropathy. A study investigating the interactions between Notch-1 and TLR4 using dorsal root ganglion (DRG) from diabetic neuropathic pain rats and cultured DRG neurons (induced by high glucose challenge) reported that high glucose concentration not only increased mRNA levels of Notch-1, Hes-1 and TLR4, but also increased protein expression of NICD-1 and TLR4 in rat DRG neurons. Incidentally, the percentage of NICD1-immunoreactive (IR) and TLR4-IR neurons in DRG cultures were found to be increased after high glucose trauma. However, the aforementioned changes were partially reversed by inhibiting either the Notch1 or TLR4 signalling pathway. Further, the inhibition of the Notch-1 or TLR4 signalling pathway decreased TNF-α levels in DRG neurons from diabetic neuropathic rats; therefore, it can be said that inhibition of either the Notch-1 signalling pathway or the TLR4 signalling pathway may improve mechanical allodynia and thermal hyperalgesia thresholds in diabetic neuropathy [206].

## 11. Developmental signalling pathways in other miscellaneous neurological abnormalities

The role of embryonic signalling pathways in AD, PD, ALS and diabetic neuropathy has been well documented. In addition, these signalling pathways play a major role in the progression of other miscellaneous brain disorders (table 1). In this section, we present the role of the embryonic signalling pathways and associated components in such disorders. In focal cerebral ischaemia, the Notch downstream target gene Hes-5 is transiently downregulated [134,218]. Strong evidence suggests that Hes-5 gene regulation can either differ between global and focal ischaemia or may be independent of Notch activation in ischaemia. Further, it is known that hypoxic stress upregulates Notch signalling primarily through the binding of HIF-1α to NICD [219]. Interestingly, NICD helps HIF-1α localize and bind to Notch-responsive promoters. Subsequently, the complex enhances the transcription of a range of genes in cerebral ischaemia. Although the fundamental mechanism driving this process is not well studied in cerebral ischaemia, it has been observed that blocking the Notch signalling pathway with GSIs confers neuroprotection and generate an anti-inflammatory ambience in focal cerebral ischaemia, thereby suggesting a potential role of the Notch signalling pathway in ischaemic damage [134] Moreover, delayed activation of the Notch cascade (through infusion of the ligand Dll4) in the lateral ventricle of adult rats has no significant effect on the infarct size, although it improves the motor skills over 45 days [220]. One possible explanation for these observations may be that Notch signalling contributes to ischaemic cell death in the acute phase of cerebral ischaemia, but facilitates neurogenesis in the later phase.

**Table 1.** Neurological disorders and their corresponding clinically specific Wnt/β-catenin, Notch and Shh functional interactions. PS-1, presenilin 1; Aβ, amyloid beta; Dkk1, dickkopf-related protein 1; ApoEε4, apolipoprotein E; LRP6, low-density lipoprotein receptor-related protein 6; BBB, blood–brain barrier; AβPP, amyloid β protein precursor; MPTP, 1-methyl-4-phenyl-1,2,3,6-tetrahydropyridine; DA, midbrain dopamine; GSK-3β, glycogen synthase kinase-3β; SNpc, substantia nigra pars compacta; SVZ, subventricular zone; NO, nitric oxide; NSAIDs, non-steroidal anti-inflammatory drugs; VM, ventral midbrain; NPCs, neural stem/progenitor cells; PARK, parkinson's gene; FOXC1, forkhead box C1; NEURG2, neurogenin 2; SPRY1, sprouty RTK signalling antagonist 1; CTNNB1, catenin beta 1; LRRK2, leucine-rich repeat kinase 2; HERC2, E3 ubiquitin protein ligase; NEURL4, neuralized E3 ubiquitin protein ligase 4; MN, motor neurons; IFN-γ, interferon gamma; HIF-1α, hypoxia-inducible factor 1-alpha; mHtt, mutant huntingtin.

s. no.	neurological disorder	signalling pathway	interaction partners	functional implications	references
1	Alzheimer's disease (AD)	Wnt/catenin pathway	PS-1 and β-catenin	AD patients carrying PS-1-inherited mutations has reduced levels of β-catenin	[145]
			Aβ and Dkk1	toxic Aβ inhibits canonical Wnt signalling and responsible for activating Dkk1, leading to synaptic loss	[207]
			ApoE*ε*4, Wnt and LRP6	ApoEε4 inhibits canonical Wnt signalling and LRP6 activity, thereby causing AD	[159,160]
			Wnt and BBB	BBB dysfunction as a result of impaired Wnt/β-catenin signalling contributes to the neurodegeneration characteristic of AD	[164]
		Notch signalling pathway	Notch proteins, PSs and AβPP	Notch proteins interact with PSs and AβPP	[133]
		Shh signalling pathway	Aβ and Shh	toxic Aβ interrupts canonical Shh signal transduction	[137,141]
			Ptch and Gli-1	brain tissue samples from AD patients and AβPP mutant transgenic mice has reduced levels of Ptch and Gli1	[142]
			Ptch-1	enhanced expression of AICD might contribute towards increased levels of Shh receptor Ptch-1	[208]
2	Parkinson's disease (PD)	Wnt/catenin pathway	Wnt-1 and MPTP	MPTP injury increases Wnt-1 expression	[88]
			Wnt-1 and MPTP	DA neuroprotection is mediated by Wnt-1 *in vivo* and *in vitro*	[88]
			DKK1, β-catenin and GSK-3β	Dkk1 i.c.v. in intact SNpc evokes DA neuronal death, decreases β-catenin and upregulates GSK-3β	[88]
			GSK-3β	DA neuroprotection is mediated by GSK-3β inhibition	[88]
			GSK-3β	neurogenesis in SVZ is provoked by using GSK-β antagonists	[88]
			NO antagonists, NO NSAIDs and Wnt/β-catenin	increased expression of Wnt/β-catenin signalling is mediated by NO antagonists, NO NSAIDs and antioxidant	[88]
			chemokine	chemokine-activated astrocytes express Wnt-1	[88]
			Wnt-1 and VM NPCs	astrocyte-derived Wnt-1 induce neurogenesis from adult SVZ and DA neurogenesis from VM NPCs	[88]
			Wnt-1	Wnt-1 expression is downregulated in aged VM astrocyte linked with microglia overactivation	[88]
			NO-flurbiprofen and GSK-3β inhibitors	NO-flurbiprofen and GSK-3β inhibitors reverse Wnt/β-catenin inhibition in NPCs	[88]
			PARK and Wnt/β-catenin	PARK genes mutation in PD is linked with impaired Wnt signalling	[128]
			MPP^+^, FOXC1, NEURG2, SPRY1 and CTNNB1	treatment with MPP^+^ to DA neuron causes epigenetic disregulation of Wnt-associated genes such as, FOXC1, NEURG2, SPRY1 and CTNNB1	[172]
		Notch signalling pathway	Notch, LRRK2, HERC2 and NEURL4	impairment in Notch signalling associated with mutated LRRK2-associated protein causes impaired endosomal trafficking and lead to PD progression	[173]
		Shh signalling pathway	Shh	interruption in the non-cell-autonomous variant of Shh emanating from DA neurons shows symptoms indicative of Parkinson's such as degeneration of dopaminergic, cholinergic and GABAergic neurons of the mesostriatal circuit and motor deficits	[174]
3	amyotrophic lateral sclorosis (ALS)	Wnt/catenin pathway	Wnt-2, Wnt-7a and GSK-3β	differential expression of the Wnt signalling components including Wnt-2, Wnt-7a and GSK-3β are responsible for MN impairment ALS mice	[176]
		Notch signalling pathway	Jagged-1	aberrant Notch signalling activation is associated with ALS pathogenesis, both in sALS patients and SOD1G93A mice	[185]
		Shh signalling pathway	Shh	Shh remains active in terminally differentiated motor neurons, enhances proliferation and differentiation of motor neuronal lineages, and increases the fraction of ciliated motor neurons	[209]
			retinoic acid (RA) and Shh	retinoic acid and Shh operate together to caudalize and ventralize motor neuron progenitors	[210]
			transcription factors (Shh, Pax3, Pax7, MSX1, MSX2, BMP4 and BMP7)	modulate the differentiation of sensory and motor neurons	[211]
4	multiple sclorosis (MS)	Shh signalling pathway	transcription factors (Pax6, Dlx2, Nkx2.1, Nkx2.2, Olig1 and Olig2)	differentiation of oligodendrocytes, myelin-forming cells enhances specification of oligodendrocyte progenitors	[212]
			Shh	Shh along with its receptors promotes BBB formation, provides an anti-inflammatory balance to the immune responses targeting the CNS	[56]
			IFN-γ and Shh	IFN-γ disturbs the Shh-induced differentiation of neural progenitors by lowering the expression levels of the transcription factor Gli1; on the contrary, IFN-γ can also upregulate Shh expression in astroglial and neural stem cells	[213]
5	traumatic brain injury (TBI)	Wnt/catenin pathway	NG2+ progenitor cells and β-catenin	β-catenin signalling in proliferating NG2+ progenitor cells of the cortex	[88]
			Wnt-5a/Fzd-2	Wnt-5a/Fzd2 is increased in ipsilateral hippocampus	[88]
			lithium, Wnt and GSK-3β	lithium inhibits GSK-3β, ameliorates neurodegeneration, and improves learning and memory	[214]
			LRP6, β-catenin and GSK-3β	LRP6, β-catenin and GSK-3β are speedily and transiently affected after TBI	[88]
6	stroke	Wnt/catenin pathway	Wnt-3a	lentivirus expressing Wnt-3a injection in SVZ and Stra induce functional recovery	[88]
			HIF-1α via Wnt	HIF-1α via Wnt is proneurogenic in stroke	[88]
			Wnt-1	Wnt-1 curtails cerebral infarction and ameliorates neurological recovery	[88]
			Dkk1 and Wnt/β-catenin	Dkk1 counteracts E2-induced Wnt/β-catenin signalling and post-ischaemia protection	[88]
			Dkk1	Dkk1 expression is upregulated in cerebral ischaemia rodent model	[88]
			GSK-3β	GSK-3β inhibitors alleviate neuron death	[88]
7	Huntington's disease (HD)	Wnt/catenin pathway	Armadillo/β-catenin, mHtt	reducing canonical Wnt signalling pathway confers protection against mHtt toxicity in *Drosophila*; mHtt has been reported to alter the stability and levels of β-catenin, a key molecule in cell adhesion and signal transduction in Wnt/β-catenin pathway	[215]
8	Alagille syndrome	Notch signalling pathway	Jagged-1	mutations in the Notch ligand Jagged-1 causes Alagille syndrome	[216]
9	CADASIL and dementia	Notch signalling pathway	Notch-3	mutations in the gene encoding the Notch-3 receptor cause CADASIL	[217]
10	cortical dysplasia	Notch signalling pathway	PS-1 and Notch-1	mutation in PS-1 gene develops global cortical dysplasia characterized by over migration of cortical plate neurons	[217]
11	holoprosencephaly	Shh signalling pathway	Shh	genetic defects in Shh signalling and teratogens such as cyclopamine that selectively inhibit Shh signalling can cause severe developmental abnormalities in the nervous systems of animals and humans	[47]
12	brain tumours	Notch signalling pathway	Delta-1 and Jagged-1 and -2	abnormal expression of Notch ligand (Delta-1 and Jagged-1 and -2) from human tumours of glial origin has been reported in brain tumours including, glioblastoma multiforme and anaplastic astrocytoma	[217]
13	hereditary leukodystrophy	Shh signalling pathway	Transcription factors (Pax6, Dlx2, Nkx2.1, Nkx2.2, Olig1 and Olig2)	differentiation of oligodendrocytes, myelin-forming cells, enhances specification of oligodendrocyte progenitors	[212]
14	schizophrenia	Shh signalling pathway	Transcription factors (Pax6, Dlx2, Nkx2.1, Nkx2.2, Olig1 and Olig2)	differentiation of oligodendrocytes, myelin-forming cells, enhances specification of oligodendrocyte progenitors	[212]
15	Klippel–Feil syndrome (KFS)	Shh signalling pathway	Transcription factors (Shh, Pax3, Pax7, MSX1, MSX2, BMP4 and BMP7)	modulate the differentiation of sensory and motor neurons	[211]
16	spinal muscular atrophy (SMA)	Shh signalling pathway	retinoic acid and Shh	retinoic acid and Shh operate together to caudalize and ventralize motor neuron progenitors	[210]
17	experimental autoimmune encephalomyelitis (EAE)	Shh signalling pathway	IFN-γ and Shh	IFN-γ disturbs the Shh-induced differentiation of neural progenitors by lowering the expression levels of the transcription factor Gli1; on the contrary, IFN-γ can also upregulate Shh expression in astroglial and neural stem cells	[213]

During CNS myelination, axonally expressed Jagged1 prevents the differentiation of oligodendrocyte precursor cells particularly through Notch-1 and Hes-5, and thereby plays a key role in regulating the timing of differentiation and myelination of oligodendrocyte precursor cells [221–223]. This mechanism accounts for the failure of differentiation of oligodendrocyte precursor cells in chronic MS [224]. In fact, oligodendrocyte precursors fail to mature in presence of inhibitory signals within the inflammatory milieu in the CNS of patients with MS [224]. Further, GSI-mediated inhibition of the Notch signalling pathway in oligodendrocytes of mice with experimental autoimmune encephalomyelitis drastically speeds up clinical recovery, promotes remyelination and limits axonal damage [225]. A recent study by Seifert *et al.* [226] in a T-cell and antibody-driven model of inflammatory demyelination reported that Notch-1 is most abundant in oligodendrocytes, especially within the lesions in shadow plaques. In addition, the group reported widespread high levels of Notch-1 and Jagged1 expression in demyelinating and remyelinating lesions in astrocytes, macrophages and axons. Moreover, ethanol exposure during embryogenesis can disturb the proliferation of radial glial cells, and thereby reduce the radial glial progenitor pool, leading to a decrease in the number of astrocytes and neurons. In addition, prenatal ethanol exposure can limit the number of progenitor cells derived from neurospheres and underregulate the expression levels of activated Notch-1 protein, suggesting that aberrations in the Notch-1 cascade may drive the neurodegenerative damage in the brain after prenatal ethanol exposure. Overall, these observations signify that the Notch protein may be expressed during both CNS remyelination and demyelination events. Therefore, the Notch-1 signalling pathway and its components hold therapeutic promise in NDDs, which is characterized by abnormalities affecting neuronal survival, impaired plasticity and reduced arborization [227,228].

Alagille syndrome, an NDD characterized by mental retardation, is caused by mutations in the Jagged1 protein that leads to impaired activation of the Notch signalling pathway [216]. In addition, mutations in the gene encoding the Notch-3 receptor are associated with disorders such as cerebral autosomal dominant arteriopathy and leukoencephalopathy, which results in recurrent strokes, progressive vascular dementia, migraines, psychiatric disturbances and pseudobulbar palsy [217]. A recent study by Ding *et al.* [229] reported that postnatal dysfunction of Notch signalling disturbs dendrite development of adult-born neurons in the hippocampus and contributes to memory loss and brain impairment. Moreover, PS-1-deficient mice exhibit strong features reminiscent of global cortical dysplasia, which is characterized by the migration of cortical plate neurons. This can be linked to irregularities in the distribution of Notch1, especially in the Cajal–Retzius neurons and cortical plate neurons, which are responsible for facilitating radial neuronal migration [217]. Finally, the activation of the Notch signalling pathway has been attributed to the development of neuropathic pain [230], which is caused by dysfunction or damage of nerve fibres present in the peripheral nervous system (PNS) or CNS. Nevertheless, the precise mechanism behind neuropathic pain is extremely complicated and hence, remains a matter of further study [230].

Several studies have reported a possible link between the Wnt signalling pathway and autism. Mouse models expressing mutant Dvl1 and Dvl3 show reduced expression of β-catenin, which facilitates premature deep layer neurogenesis of neural progenitors in specific regions of the brain during embryogenesis. This ultimately exerts a harmful effect on the formation of neural connections in the prefrontal cortex in the future, as represented by severe deficiencies in the brain size and social behaviour of full-grown adults [231]. However, this deficit can be overturned by administering a GSK-3 inhibitor that helps in reactivating the canonical Wnt signalling pathway *in utero*. Fragile-X-linked mental retardation protein (FMRP) plays a role in driving autism-like behaviour. In the brain of Fmr1 (FMRP gene) knockout mice, FMRP was found to be a negative regulator of Wnt-2 mRNA expression. Incidentally, patients with Fragile-X syndrome have reduced expression levels of Wnt-7, and hence limited activation of the Wnt/β-catenin signalling pathway [172,232–240]. Studies have shown that patients with schizophrenia display altered GSK-3 activity, as well as amplified expression levels of Wnt-1 that lead to synaptic rearrangement and plasticity [240,241]. In addition, an array of single nucleotide polymorphisms in Fzd3 has been reported to be associated with vulnerability to schizophrenia [242,243]. Moreover, the relevance of the Wnt signalling pathway in depression, bipolar disorder, epilepsy and seizures has been studied by various research groups [244,245]. Overall, these studies indicate the significance of the Wnt signalling pathway and its associated components in the pathogenesis of neuronal diseases and brain trauma and can serve as potential targets for therapeutic intervention in the near future.

## 12. Developmental signalling pathways as therapeutic drivers in neurological abnormalities

Several studies have reported the direct therapeutic implication of embryonic signalling pathways in neurological disorders. For instance, a study indicated that Wnt-5a activation prevents synaptic loss triggered by toxic Aβ peptides and helps in monitoring the decrease in the amplitude of excitatory postsynaptic currents triggered by the same peptides. Strong evidence suggests that Wnt-5a modulates or rather balances the synaptic strength through the Wnt/PCP–JNK signalling axis primarily by limiting the reduction of PSD-95 postsynaptic clusters [246]. Moreover, Wnt-5a assists in the trafficking and localization of γ-aminobutyric acid (GABAA) and NMDA receptors to the neuronal surface, which eventually helps in the growth of dendritic spines. Moreover, Wnt-5a protects the neuronal mitochondria from toxic Aβ oligomers by triggering the Wnt/Ca^2+^ axis [247–250]. Similar studies have found that the Shh pathway is crucial in mediating cerebral angiogenesis that increases blood flow and leads to favourable outcomes in stroke and chronic NDDs such as AD [251]. Moreover, specific AD-related studies reported that the activation of the Shh signalling pathway conferred protection to the hippocampal neurons against the toxic effects of Aβ by inducing the BDNF expression and promoting autophagy [252–257]. Additionally, Shh signalling can play neuroprotective roles in cerebral ischaemia by inhibiting excitotoxicity, oxidative stress, neuroinflammation and apoptosis of neurons. The Shh/PI3K/AKT pathway may be one of the possible underlying mechanisms as reported by Liu *et al.* [258]. The activation of the Shh signalling pathway exerts protective effects by enhancing the expression of antioxidant enzymes such as SOD and glutathione peroxidase (GSH-Px), reducing apoptotic genes such as P53 and caspase-3, and enhancing the anti-apoptotic genes such as Bcl-2 and BDNF. In addition, Shh is engaged in protecting neurons against NMDAR-dependent excitotoxicity [259]. Finally, Notch has been found to be extremely crucial in intrastriatal transplantation therapy for ischaemic stroke. Studies have reported that the activation of the Notch signalling pathway helps in hastening endogenous regeneration of the hippocampal neurons. Further, the Notch signalling activity facilitates increased arteriogenesis in a rat model of middle cerebral artery occlusion (MCAO) stroke [259–263]. Nevertheless, in addition to the few interactions described in this section, there are many more mechanisms through which embryonic signalling pathways exert therapeutic effects (table 2), and thus warrant more in-depth study.

**Table 2.** Therapeutic roles of developmental signalling pathways and their components in neurological abnormalities. PSD-95, postsynaptic density protein 95; GABAA, γ-aminobutyric acid; NMDA, *N*-methyl-d-aspartate receptor; LTP, long-term potentiation; PPAR-α, peroxisome proliferator-activated receptor alpha; Ach, acetylcholine; PKC, protein kinase C; BDNF, brain-derived neurotrophic factor; NSCs, neural stem cells; SOD, superoxide dismutase; GSH-Px, glutathione peroxidase; Bcl-2, B-cell lymphoma 2; NMDAR, *N*-methyl-d-aspartate receptor; 6-OHDA, 6-hydroxydopamine.

s. no.	signalling component	signalling pathways	clinical significance	associated abnormalities	references
1	Wnt-5a	Wnt/PCP/JNK pathway	it can prevent the decrease in the amplitude of excitatory postsynaptic currents induced by Aβ oligomers and thus prevent the synaptic loss triggered by Aβ; it also prevents the decrease in the PSD-95 postsynaptic clusters through the Wnt/PCP–JNK pathway and thus assists in modulating synaptic strength	Alzheimer's disease	[246]
2	Wnt-5a	Wnt/Ca^2+^ pathway	it provokes the trafficking of GABAA and NMDA receptors to the neuronal surface, helps in the growth of dendritic spines and protects neuronal mitochondria from toxic Aβ oligomers through triggering Wnt/Ca^2+^ pathway		[247–250]
3	small Wnt molecules	Wnt/catenin pathway	small Wnt molecules can activate both canonical and non-canonical Wnt signalling *in vivo* to increase memory cognition in adult mice and reverses cognitive impairments and LTP in the AΒPPswe/PS-1 transgenic model of AD		[264,265]
4	Wnt	anti-inflammatory signalling pathways	activation of numerous signalling modules including PPAR-α and *γ*, the nicotinic and muscarinic ACh receptors, antioxidants and anti-inflammatory pathways in association with Wnt signalling supports the neuroprotective role of the Wnt signalling cascade in AD		[266–268]
5	Wnt	Wnt/catenin pathway	Wnt signalling plays a crucial role in learning and memory processes through modulation of synaptic structure and function and thus curtails the cognitive deficit associated with AD		[269]
6	Wnt	Wnt/catenin pathway	it helps in maintaining synaptic strength in the CNS by regulating the translocation of a subset of AChRs to synapses and thus assists in curtailing synaptic loss associated with AD		[270,271]
7	Wnt	Wnt/catenin pathway	enhanced expression of Wnt-1 protects neurons against Aβ-mediated oxidative stress, and oxidative DNA damage in primary hippocampal murine neurons and thus confers neuroprotection against oxidative stress in AD		[272,273]
8	Wnt	Wnt/β-catenin pathway	Wnt pathway regulates PKC activity and increased activity of PKC causes downregulation of Aβ production		[274]
9	Shh	Shh pathway	Shh can protect hippocampal neurons against Aβ toxicity by provoking BDNF production and thus shows a neuroprotective effect in experimental models of AD		[252–254,256]
10	Shh	Shh pathway	it might bolster neuronal resistance in degeneration by increasing autophagy; impaired autophagy is accountable for agglomeration of cytotoxic proteins (Aβ and p-tau) and dysfunctional mitochondria in AD; enhancement in the activity of autophagy by bolstering mitochondrial bioenergetics can improve AD-like Aβ and p-tau pathologies in a mouse model of AD		[255,257]
11	Wnt-1	Wnt/β-catenin pathway	the Wnt-1-augumented neuroprotection is closely related to the astroglial response to oxidative stress and inflammation upon injury and requires Fzd-1 receptor and β-catenin stabilization coupled to GSK-3β inhibition to promote mDAergic neuron survival. Reactive astrocytes also upregulate Wnt/β-catenin signalling and thus contribute to neuroprotection	neuroinflammation and Parkinson's disease	[66,241]
12	Wnt	Wnt/β-catenin pathway	Wnt signalling at the neuroimmune interface plays a crucial role in the regulation of neuroprogenitors, post-mitotic neurons and microglial cell functions in PD	Parkinson's disease	[66,241]
13	Notch	Notch pathway	Notch signalling and its associated components can exert neuroprotection via enhancing endogenous neuroregeneration and brain arteriogenesis following stroke	stroke	[275,276]
14	Notch	Notch pathway	Notch signalling and its associated components can induce neuronal expansion and differentiation following stroke and also helps in the maintenance, proliferation, and differentiation of NSCs in the developing brain	stroke	[259–261]
15	Notch	Notch pathway	increased expression of Notch signalling and associated components may facilitate intrastriatal transplantation therapy for ischaemic stroke by advancing endogenous regeneration in the hippocampus; interestingly Notch signalling activity may also facilitate increased arteriogenesis in a middle cerebral artery occlusion stroke rat model	ischaemic stroke	[262,263]
16	Shh	Shh pathway	topical application or intrathecal administration of Shh to the brain surface above the cerebral infarct region leads to an improved functional outcome, diminished neuronal degeneration and enhanced neurogenesis in a rat model of focal ischaemic stroke	focal ischaemic stroke	[277,278]
17	Shh	Shh pathway	Shh signalling may induce cerebral angiogenesis which could lead to enhanced functional outcomes in stroke and chronic neurodegenerative disorders such as AD; reduced blood flow to affected brain regions, including the hippocampus is one of the characteristic features of AD	stroke and chronic neurodegenerative disorders	[251]
18	Shh	Shh/PI3K/AKT pathway	Shh signalling pathway and its components exert a protective effect in cerebral ischaemia via increasing the expression of antioxidant enzymes such as SOD and GSH-P_X_, decreasing apoptotic genes such as P53 and caspase-3, and increasing the expression of anti-apoptotic genes such as Bcl-2 and BDNF; additionally, Shh also helps protecting neurons against NMDAR-dependent excitotoxicity	cerebral ischaemia/ischaemic stroke	[76]
19	Shh	Shh pathway	Shh and its components promote the survival of fetal dopaminergic neurons and protect cultures of fetal midbrain dopaminergic neurons from the toxic effects of MPP^+^, which selectively injures nigral dopaminergic neurons; Shh also reduces behavioural deficits induced by intrastriatal 6-OHDA lesion *in vivo* as suggested by numerous studies	Parkinson's disease	[279]

## 13. Therapeutic application of neuroprotectants and their effects on developmental signalling pathways

In the preceding sections, we have extensively discussed the role of embryonic signalling pathways both in maintaining normal cellular homeostasis and in driving the prognosis of several human neurological disorders. Incidentally, the deregulations have not only been observed with respect to the core signalling components but also with other accessory components and pathways. Therefore, there exists a strong incentive to explore the therapeutic potential of these components in disorders such as AD, PD, ALS, stroke, traumatic brain injury (TBI) and ischaemic strokes. Recently, studies have evaluated several compounds with the ability to target the interactome of the embryonic signalling cascade in the nervous system and the process alleviates or blocks the physiological deregulations that can be associated with neuronal damage. This includes compounds such as antioxidants, biomolecules and non-steroidal anti-inflammatory drugs (NSAIDs), all of which have specific targets (tables 3 and 4). Nevertheless, few compounds have reached the stage of clinical trials, thereby warranting further research to establish the precise mechanism of action of the compounds. In the brain, the normal functioning of embryonic signalling pathways is imperative for neuronal survival because they play an important role in the formation and definition of the plasticity of neuronal circuits in the CNS. Recently, the deregulations associated with developmental cues in neurological disorders are gaining increased recognition, thereby making them an interesting area of research in neurosciences. The results can be well seen in cases of severe neurological traumas such as those involving thought, memory, language, behaviour and planning. Therefore, it is crucial to minimize the deregulation of the signalling components with the help of inhibitors or activators to revert to normal neuronal homeostasis behaviour post-trauma.

**Table 3.** Various drugs and biomolecules and their targets to modulate defective developmental signalling pathways in Alzheimer's disease. PP2A, protein phosphatase 2A; GSK-3β, glycogen synthase kinase-3β; Wif-1, Wnt inhibitory factor 1; Dkk, Dickkopf-related protein 1; TCF/LEF, T-cell factor/lymphoid enhancer factor; α7-nAChRs, homomeric α7 nicotinic acetylcholine receptors; AChE, acetylcholinesterase; PPARs, peroxisome proliferator-activated receptors; NSAIDs, non-steroidal anti-inflammatory drugs; PARP, poly ADP ribose polymerase; ROS, reactive oxygen species.

s. no.	compounds	signalling pathway	signalling molecules/factors	functional Implications	disease	references
1	fluoxetine (FLX)	Wnt/β-catenin	PP2A, GSK-3β, β-catenin, AβPP and Aβ	fluoxetine treatment increases the activity of PP2A; the activation of PP2A, caused by fluoxetine plays a positive role in raising the level of active β-catenin, and delivers a negative impact on GSK-3β activity in the hippocampal tissue thereby showing protective effects on neuron synapse	Alzheimer's disease	[280]
2	curcumin	Wnt/β-catenin	GSK-3β, Wif-1, Dkk, TCF/LEF and cyclin D1	curcumin nanoparticles increase neuronal differentiation by activating the Wnt/β-catenin pathway, involved in the regulation of neurogenesis; these nanoparticles help in enhancing nuclear translocation of β-catenin, decrease GSK-3β levels, and increase promoter activity of the TCF/LEF and cyclin D1 and thus reverse learning and memory impairments in an Aβ induced rat model of AD		[281]
3	AF267B	Wnt/β-catenin	GSK-3β, Aβ and tau	chronic AF267B (a specific agonist of M-1 muscarinic receptor) administration in the 3 × Tg-AD model causes to rescue cognitive deficits in a spatial task and curtail Aβ and tau pathologies in the hippocampus due to GSK-3β inhibition and Wnt signalling activation		[282]
4	NSAIDs	Wnt/β-catenin	β-catenin and Aβ	NSAIDs, α7-nicotinic acetylcholine receptors (α7-nAChRs), an inhibitor of acetylcholinesterase (AChE), and peroxisome proliferator-activated receptors (PPARs) are involved in the activation of Wnt signalling pathway and protect against Aβ toxicity		[283]
5	IBU-PO	Wnt/β-catenin	GSK-3β, β-catenin, AβPP and Aβ	IBU-PO combines an NSAID (Ibuprofen) and a cholinesterase (ChE) inhibitor (Octyl-pyridostigmine) to inhibit GSK-3β function and stabilize β-catenin, reverting the silencing of the Wnt signalling caused by Aβ toxicity and GSK-3β overexpression		[284]
6	Huperzine A	Wnt/β-catenin	GSK-3β and β-catenin	a reversible and selective inhibitor of AChE, Huperzine A (HupA) activates Wnt signalling via GSK-3β inhibition and stabilizes the level of β-catenin and reduces amyloidosis in the AD brain		[285]
7	troglitazone	Wnt/β-catenin	β-catenin and Aβ	the PPARγ agonist troglitazone prevent β-catenin destabilization induced by Aβ and induces translocation of cytoplasmic β-catenin to the nucleus, resulting in protection of hippocampal neuron morphology in cells exposed to Aβ		[286]
8	nicotine	Wnt/β-catenin	α7-nAChR and β-catenin	nicotine, an unselective α7-nAChR agonist prevents memory deficits and synaptic impairment in AD; also stabilizes β-catenin and prevents Aβ-induced loss of β-catenin through the α7-nAChR		[273]
9	lithium	Wnt/β-catenin	Wnt	lithium induced Wnt activation can result in cognitive improvement		[287,288],
10	rosiglitazone	Wnt/β-catenin	Wnt	rosiglitazone induced Wnt activation can result in cognitive improvement		[287,288],
11	triazine derivatives (TRZ-15 and TRZ-20)	Wnt/β-catenin	Aβ1–42, Cytochrome c, cleaved caspase-3, pGSK-3/GSK-3 and β-catenin	triazine derivatives show neuroprotective effect via activating Wnt/β-catenin signalling in rodent models of AD		[289]
12	isoalloxazine derivatives (7 m and 7q)	Wnt/β-catenin	Aβ1–42, p-tau, cleaved caspase-3, cleaved PARP levels, p-GSK-3, β-catenin and neuroD1 levels	novel multi-targeted isoalloxazine derivatives in rodent models of AD shows neuroprotective potential through activation of canonical Wnt/β-catenin signalling cascade		[290]
13	cannabidiol (CBD)	Wnt/β-catenin	PPAR*γ*, ROS, tau and AChE	CBD exerts neuroprotective effect via stimulation of PPARγ through the Wnt/β-catenin pathway to protect PC12 cells from Aβ neurotoxicity and oxidative stress, increases cell survival, diminishes mitochondrial dysfunction and ROS generation, reduce lipid peroxidation, ubiquitination of AβPP, inhibit the hyperphosphorylation of tau via GSK-3β downregulation, inhibit AChE, and stimulate the neurogenesis of the hippocampus		[291,292].

**Table 4.** Various drugs and biomolecules and their targets to modulate defective developmental signalling pathways in neurological abnormalities. SOD, superoxide dismutase; GSH-Px, glutathione peroxidase; MDA, malondialdehyde; Δψm, mitochondrial membrane potential; OHDA, 6-hydroxydopamine; H_2_O_2,_ hydrogen peroxide; ROS, reactive oxygen species; GSH, glutathione; NDDs, neurodegenerative disorders; BDNF, Brain-derived neurotrophic factor; NGF, nerve growth factor; NICD, notch intracellular domain; NSS, TBI, traumatic brain injury; DAPT, MCAO/R, middle cerebral artery occlusion-reperfusion; OGD, against oxygen-glucose deprivation; ATP, adenosine triphosphate; TFAM, mitochondrial transcription factor A; NRF-1, nuclear respiratory factor 1.

s. no.	compounds	signalling pathway	signalling molecules/factors	functional implications	diseases	references
1	curcumin	Wnt/β-catenin	Wnt-3a, c-myc, cyclin D1, SOD, GSH-Px, MDA and Δψm	curcumin enhances viability, survival and adhesion and attenuates apoptosis of deutocerebrum primary cells by triggering the Wnt/β-catenin signalling pathway in 6-OHDA PD model of rats	Parkinson's disease	[293]
2	curcumin	Notch signalling	H_2_O_2_, ROS, GSH	treatment with curcumin protects SK-N-MC cells from H_2_O_2_-induced cell death by modulation of Notch signalling pathway	neuronal cell death	[294]
3	resveratrol	Notch signalling	NA	resveratrol inhibits Notch signalling pathway to improve spinal cord injury	SCI	[295]
4	curcumin	Wnt/β-catenin	GSK-3β and β-catenin	curcumin has been reported to show neuroprotection against NDDs by activating the Wnt/β-catenin signalling pathway through inhibiting the expression of GSK-3β and inducing the expression of β-catenin and Cyclin D1	neurodegenerative disorders	[296]
5	salvianolic acid	Shh signalling	Gli1, Smo, BDNF and NGF	salvianolic acid provokes functional recovery and neurogenesis via activation of Shh after stroke in mice; it also increases the expression of Shh and Ptch along with heightened nuclear translocation of Gli1 in the peri-infarct region, thereby causing robust production of BDNF and nerve growth factor	stroke	[297]
6	resveratrol	Shh signalling	Shh, Ptch-1, Smo and Gli-1	resveratrol decreases cerebral ischaemic injury and improves neurological function by upregulating Shh signalling pathway	ischaemic stroke and cerebral ischaemic injury	[298]
7	SAG	Shh signalling	Smo and NSCs	SAG being a chlorobenzothiophene-containing Shh pathway agonist, binds to the Smo heptahelical bundle in a fashion similar to the Smo inhibitor cyclopamine and enhances the survival of nascent NSCs extracted from both SVZ and SGZ in the ischaemic brains; SAG administration further also improves cognitive function and locomotor activity in ischaemic brains	ischaemic stroke	[299]
8	cerebrolysin	Shh signalling	NA	Shh pathway mediates cerebrolysin-enhanced neurogenesis and white matter remodelling and improves functional recovery in rats after stroke	stroke	[300]
9	crocin	Notch signalling	NICD and Hes-1	crocin-induced activation of Notch signalling and protects against TBI-induced inflammation and apoptosis; it also improves neurological severity score (NSS) and brain oedema, decreases microglial activation and release of several pro-inflammatory cytokines, and decreases cell apoptosis in TBI mice	traumatic brain injury	[301]
10	DAPT	Notch signalling	Notch, Hes-1 and Hes-5	Notch inhibitor DAPT reduces oxidative stress and apoptosis after acute craniocerebral injury via inhibiting Notch, Hes-1 and Hes-5 activity and also improves neurological and cognitive function	Acute craniocerebral injury	[302]
11	nicotine	Wnt/β-catenin	α7-nAChR	nicotine treatment shows α7 nicotinic receptor-mediated neuroprotection of dopaminergic neurons in a mouse PD model via Wnt/β-catenin signalling	Parkinson's disease	[303]
12	lithium	Wnt/β-catenin	β-catenin	lithium shows neuroprotection against ALS via activating downstream components of the Wnt signalling pathway *in vivo*, leading to an increase of the β-catenin protein	amyotrophic lateral sclerosis	[304]
13	irisin	Notch signalling	NICD, Notch-1 and Hes-1	irisin shows neuroprotection against cerebral ischaemia/reperfusion injury via Notch signalling pathway	cerebral ischaemia/reperfusion injury	[305]
14	minocycline	Notch signalling	Notch-1	minocycline attenuates the development of diabetic neuropathy by inhibiting spinal cord Notch signalling in the rat	diabetic neuropathy	[306]
15	purmorphamine	Shh signalling	Smo	treatment of mice with the Smo agonist purmorphamine beginning shortly after experimental focal cerebral ischaemia improves functional outcome and lessens brain damage and neuroinflammation in a stroke model	ischaemic stroke	[307]
16	purmorphamine	Shh signalling	Smo and PI3K/AKT	activation of Shh signal by Purmorphamine, in a mouse model of PD protects dopaminergic neurons and attenuates inflammatory response by mediating PI3K/AKT signalling pathway	Parkinson's disease	[308]
17	isoflurane	Notch signalling	Notch-1, Hes-1 and NICD	neuroprotective effects of isoflurane preconditioning in a murine transient global cerebral I/R model are mediated by the pre-activation of the Notch signalling pathway; isoflurane preconditioning accelerates the expression of Notch-1, Hes-1 and NICD after ischaemic-reperfusion	cerebral ischaemia	[309]
18	sevoflurane	Notch signalling	NICD, Hes-1 and Hes-5	sevoflurane preconditioning shows neuroprotective effects against transient cerebral ischaemic injuries via activation of canonical Notch signalling pathway in mice	cerebral ischaemic injury	[310]
19	osthole	Notch signalling	Notch-1	administration of Osthole (a coumarin derivative) prevents ischaemia–reperfusion injury via activating the Notch-1 signalling pathway both *in vivo* and *in vitro* in a dose-dependent manner; it also enhances the activity of Notch-1 signalling and reduces the cerebral infarction as well as the hippocampus neuronal injury and apoptosis induced by MCAO/R in a dose-dependent manner	ischaemia–reperfusion injury	[311]
20	rosuvastatin (RSV)	Notch signalling	ROS, OGD, ATP, mtDNA,TFAM, NRF-1 and Notch-1	RSV ameliorates neurite outgrowth of cortical neurons against OGD via Notch-1-mediated mitochondrial biogenesis (increases the mtDNA content and the mRNA levels of mitochondrial transcription factor A (TFAM) and NRF-1) and functional improvement	cerebral ischaemic injury	[83]
21	simvastatin	Notch signalling	PS-1 and NICD	simvastatin increases arteriogenesis after stroke by enhancing PS-1 activation of the Notch signalling pathway	stroke	[263]

**Figure 5.**
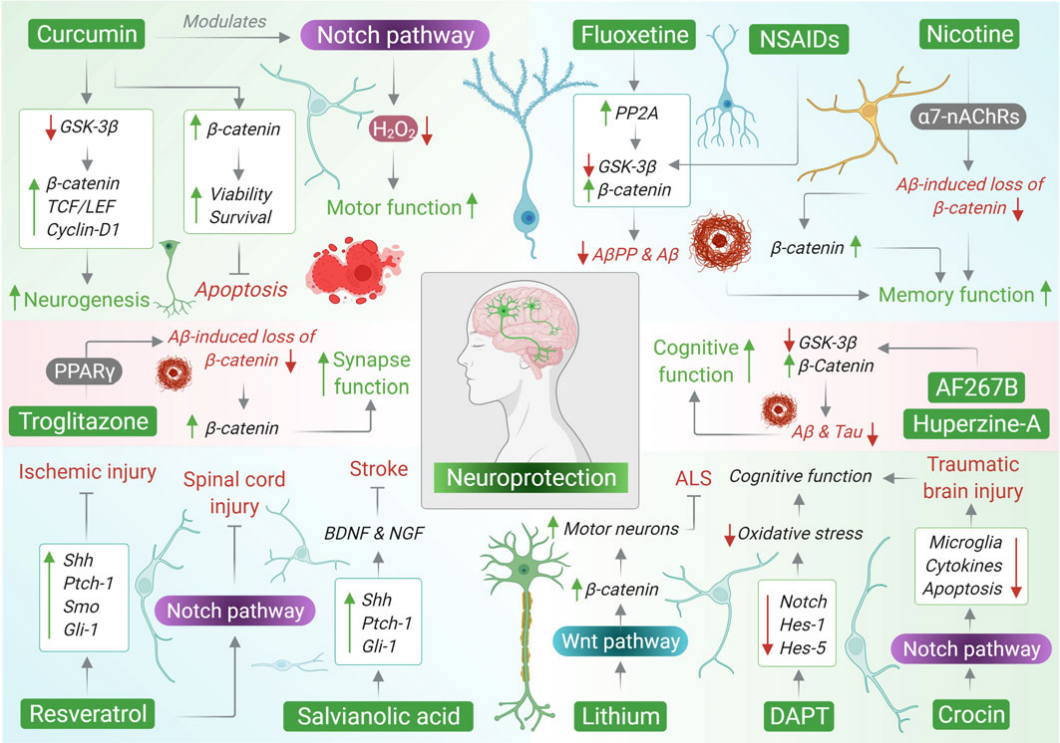
Drug- and biomolecule-mediated regulation of developmental signalling pathways in neurological disorders.

Several biological compounds are currently under consideration because of their unique ability to address cell toxicity-related effects by targeting the embryonic signalling pathways and their associated components in neurological disorders (figure 5). For instance, fluoxetine treatment has been shown to confer neuroprotection against AD, primarily by increasing the activity of protein phosphatases of type 2A (PP2A). Increased PP2A levels downregulate the GSK-3β activity in the hippocampal tissue, which helps in augmenting the level of active β-catenin. Both these changes lead to the activation of the Wnt/β-catenin signalling pathway that in turn limits AβPP cleavage and Aβ peptide generation. Further, fluoxetine treatment prevents apoptosis, as observed in 3×Tg-AD primary neuronal cell model, in addition to promoting neuroprotective ambience in the neuron synapse [280]. Nicotine, an unselective 7-nicotinic acetylcholine receptor (α7-nAChR) agonist, inhibits memory deficits and synaptic impairment in AD. Evidence shows potential crosstalk between the α7-nAChR and Wnt/β-catenin signalling pathways because nicotine stabilizes β-catenin and prevents the Aβ-induced loss-of-β-catenin through the activity of α7-nAChR [312]. Further, studies have highlighted the neuroprotective effects of NSAIDs, which target α7-nAChRs, the inhibitor of acetylcholinesterase and peroxisome proliferator-activated receptors (PPARs), primarily by activating the Wnt signalling pathway and thereby, conferring protection against Aβ-induced toxicity in AD brains [283]. Likewise, troglitazone, the PPARγ agonist, prevents changes in the Wnt signalling cascade, triggered by Aβ peptide. The activation of neuronal PPARγ prevents β-catenin destabilization triggered by Aβ and facilitates the translocation of cytoplasmic β-catenin to the nucleus, thus protecting the hippocampal neuron morphology in cells exposed to Aβ stress [286].

A study by Tiwari *et al.* [281] examined the potential of curcumin nanoparticles in reversing learning and memory impairments caused by Aβ peptides. The group studied the effects of curcumin nanoparticles in an Aβ-induced rat model and observed that curcumin nanoparticles induced neuronal differentiation by triggering a self-repair mechanism involving the Wnt/β-catenin signalling pathway. Further, these nanoparticles promoted the nuclear translocation of β-catenin, decreased the GSK-3β levels and enhanced the promoter activity of the TCF/LEF transcription factor. *In silico* molecular docking studies have revealed the possibility of interaction between curcumin and Wif-1, Dkk and GSK-3β in AD models [281]. Curcumin can increase the viability, survival and adhesion while limiting the apoptosis of deutocerebrum primary cells primarily by activating the Wnt/β-catenin signalling pathway. Further, curcumin administration increases mRNA and protein expression of Wnt-3a, β-catenin, c-Myc and cyclin D1. In addition, curcumin administration in 6-OHDA rat model of PD results in increases the levels of SOD and GSH-Px and decreases malondialdehyde and heightens mitochondrial membrane potential (Δψm) [293]. Interestingly, curcumin treatment protects neuroblastoma cell line (SK-N-MC cells) from peroxide-induced cell death by modulating the Notch signalling pathway [294], thereby suggesting the role of curcumin in modulating both the Wnt and Notch signalling pathways for neuroprotection. Studies have suggested that the canonical Wnt/β-catenin signalling pathway is mainly downregulated in AD and this downregulation is responsible for the augmentation of oxidative stress, neuroinflammation and dysregulation of the glutamatergic pathway in AD. The use of riluzole can be an interesting therapeutic strategy in AD because it specifically targets and activates the Wnt/β-catenin signalling pathway. Nevertheless, future clinical trials may reveal whether riluzole can confer beneficial effects in AD [313].

Salvianolic acid is a known antioxidant and free radical scavenger that has currently been reported to invoke neurogenesis in the CNS by activating the Shh signalling pathway after stroke. Salvianolic acid boosts the proliferation of NPCs and promotes the long-term survival of nascent neurons in the SVZ. Further, the compound upregulates both the mRNA and protein levels of Shh and Ptch and facilitates the nuclear translocation of Gli1 in the peri-infarct region, thereby resulting in the increased production of growth factors such as BDNF and NGF, which altogether helps to build a neuroprotective environment [297]. Recently, another molecule called resveratrol has exhibited neuroprotective properties, especially against ischaemic stroke through a range of processes such as anti-oxidation, anti-inflammation and anti-apoptosis. It has been argued that resveratrol substantially increases the RNA expression of the Shh signalling pathway components such as Shh, Ptch-1, Smo and Gli-1. In addition, resveratrol promotes the nuclear translocation of Gli-1, which can improve the neural condition after cerebral ischaemic injury by upregulating the Shh signalling activity [298]. The therapeutic potential of smoothened agonist (SAG) is being studied in ischaemic brains. SAG being a chlorobenzothiophene-containing Shh pathway agonist, binds to the Smo heptahelical bundle in a fashion similar to the Smo inhibitor cyclopamine and enhances the survival of nascent NSCs extracted from both the SVZ and SGZ of the ischaemic brains. In addition, SAG administration improves cognitive function and locomotor activity in ischaemic brains [299]. Finally, another compound called irisin can reduce the morphological damage and mend the neurological activities after global cerebral ischaemia–reperfusion (I/R) injury in a mouse model. Irisin has anti-apoptotic properties in the brain; it downregulates the expression levels of neuroinflammatory mediators, IL-1β and TNF-α and upregulates the expression of NICD, Notch-1 and Hes-1 both *in vitro* and *in vivo*. Hence, treatment of cells with GSI inhibitor (DAPT) leads to a complete reversal of all the morphological, neurological and biochemical changes. The results indicate that irisin potentially regulates the Notch signalling pathway that ultimately leads to the mitigation of transient global cerebral I/R injury effects in mouse models [305]. In addition to the few compounds discussed in this section, there are many molecules (table 4) that have exhibited potential or need further investigation to concretely establish their neurotherapeutic potential in a range of neural disorders.

## 14. Novel therapeutic strategies for modulating the developmental signalling pathway components

In the preceding sections, we discussed the role of neuroprotectants and their mechanism of action and disease–critical interactions for improving prognosis in patients. However, methods other than drug-mediated targeting are available to exploit embryonic signalling pathway components to curtail the disease burden. The Wnt-mediated activation of glucose metabolism has been reported to arbitrate the *in vivo* neuroprotective effects of the Wnt signalling pathway in AD. Wnt activators in any form can enhance the use of brain glucose and cognitive function, as observed in the transgenic mouse model of AD. Wnt activators help enhance glucose metabolism by activating the Wnt signalling pathway, which promotes the expression of hexokinase, phosphofructokinase and AMP-activated protein kinase. The current study highlights the neuroprotective effects of the Wnt signalling pathway in mouse models of AD at least in part through the Wnt-mediated improvements in neuronal glucose metabolism [314]. Further, some studies have suggested that Dkk3 can drive cerebral glucose metabolism or glucose uptake deficits in AD. In addition, transgenic expression of Dkk3 in the mouse model of AD was found to improve learning, memory and locomotor activity, by limiting Aβ accumulation. Moreover, transgenic Dkk3 overexpression resulted in the downregulation of GSK-3β, a known negative regulator of the canonical Wnt signalling pathway, and upregulation of PKCβ1 (a factor involved in the non-canonical Wnt signalling pathway). Overall, these observations highlight a crucial fact that the deregulation of Dkk3, GSK-3β and PKCβ1 expression may be a potential therapeutic strategy for AD [315]. The Wnt signalling pathway mediates the neuroprotective effects of neuroglobin (Ngb) by promoting neurogenesis in certain neurodegenerative conditions such as stroke. It has been noted that the enhanced activity of Ngb elicits the proliferation of NPCs, characterized by an increase in neurosphere number and size. Ngb overexpression can promote neuronal differentiation of cultured NPCs under specific differentiation conditions. In addition, injection of Lv-Ngb in the SVZ of mice after MCAO enriches the population of polysialylated neuronal cell adhesion molecule (PSA-NCAM) positive neuroblasts and neuron-specific class III β-tubulin (Tuj1) positive immature neurons, thereby facilitating neurogenesis in mice brain after stroke. There is a strong indication that the research community should consider that the pro-neurogenesis effect of Ngb overexpression could be mediated by Dvl1 upregulation and subsequent activation of the Wnt signalling pathway, resulting in increased nuclear β-catenin stabilization [316]. Finally, Norrin has well-known neuroprotective features in retinal neurons with a strong potential to limit the damaging effects of NMDA-induced retinal ganglion cell loss. Although few studies have reported, there is an indication that the neuroprotective effects of Norrin could be mediated by the activation of the Wnt/β-catenin signalling pathway and subsequent induction of neurotrophic growth factors in Müller cells [317].

Remote ischaemic preconditioning (RIPC) is a new strategy that initiates endogenous protective pathways in the brain and therefore outlines a promising therapeutic strategy against cerebral I/R injury. Moreover, RIPC was found to improve neurological scores and reduce infarct volume and neuronal apoptosis in rats subjected to I/R injury. Recently, the pre-activation of Notch-1 as part of RIPC was found to reduce cerebral ischaemia–reperfusion injury, primarily through NF-κβ crosstalk. The NF-κβ signalling pathway is a well-known downstream target of Notch-1 and helps in protecting from focal cerebral I/R injury during RIPC [318]. Similar to RIPC, cerebral ischaemic preconditioning (cIPC) performs a pivotal role in neuroprotection under conditions of Notch pre-activation. *In vivo* experiments have assessed the neuroprotective role of cIPC and found that cIPC lessens the neurological functional deficit, cerebral infarction and cellular apoptosis in the hippocampal neurons, induced by middle cerebral artery occlusion/reperfusion (MCAO/R). Nevertheless, these observations indicate that cIPC can improve neurological function in a Notch-dependant manner. Moreover, both RIPC and cIPC can upregulate the expression levels of Jagged1, Notch-1, NICD and Hes-1 proteins. Importantly, both RIPC and cIPC-induced changes in neurological function can be compromised through the activity of GSIs such as DAPT. On the other hand, OGD preconditioning can upregulate Notch-1 expression and signalling in OGD/R-treated neurons and NSCs; thus, OGD/R treatment limits neuronal death and apoptosis. Furthermore, Notch-1 pre-activation limits the percentage of cells in the G1 stage and enhances the percentage of cells in the S stage in the OGD/R-treated NSCs. Overall, the neuroprotective effects of RIPC cIPC in a MCAO/R rat model are essentially preceded by the pre-activation of the Notch signalling pathway [319].

**Figure 6.**
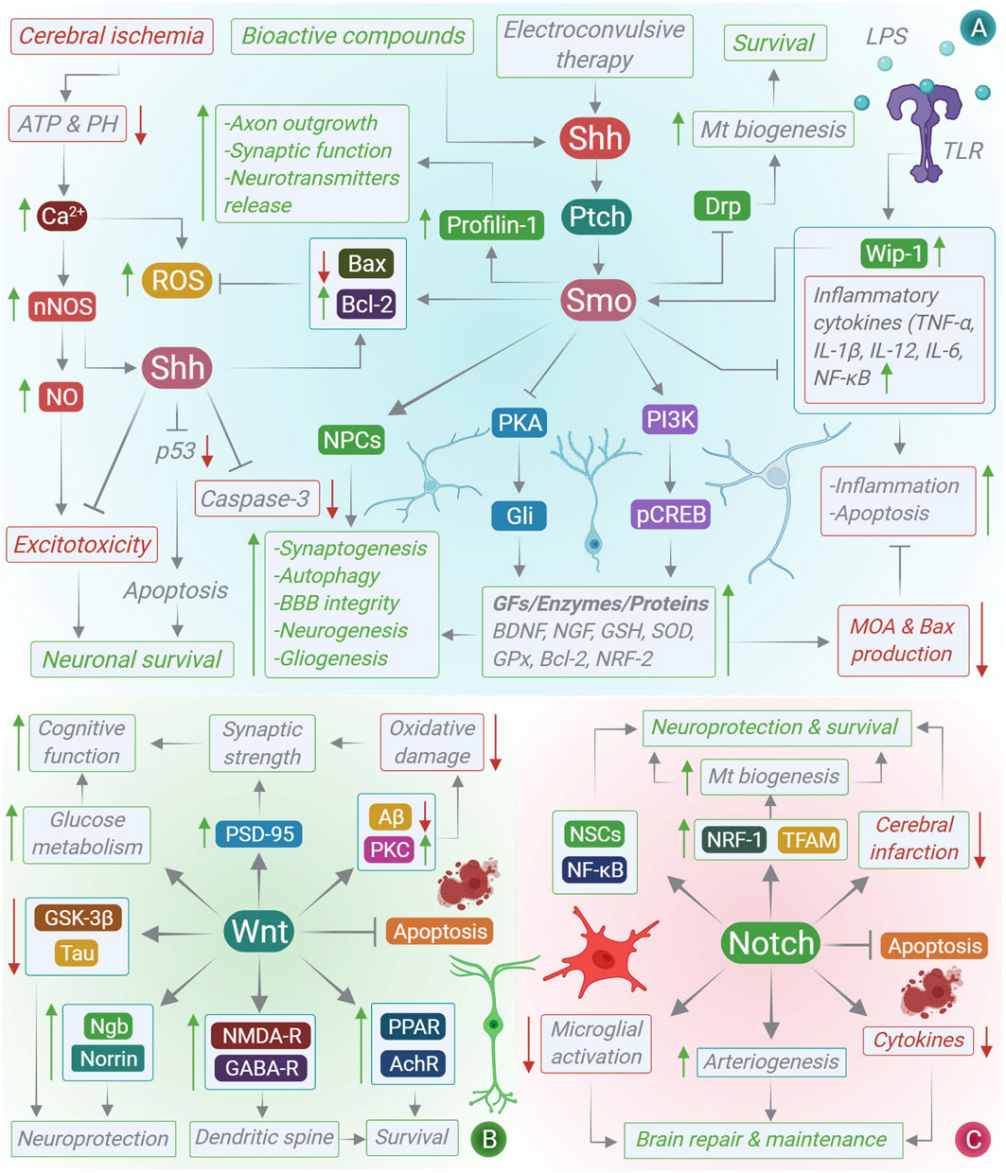
Proposed mechanistic role of the developmental signalling pathways in neuronal survival and neuroprotection. (A) The Shh signalling pathway can exert neuroprotective effects in cerebral ischaemia via the inhibition of oxidative stress, excitotoxicity, inflammation, and apoptosis of neurons. The Shh/PI3K/AKT pathway may be the underlying mechanism. Shh signalling pathway activation exerts protective effects by increasing antioxidant enzymes such as SOD, GSH-P_X_, decreasing apoptotic genes such as P53 and caspase-3, and increasing anti-apoptotic genes such as Bcl-2 and BDNF. Shh is also involved in protecting neurons against NMDAR-dependent excitotoxicity. It can also help in BBB function, autophagy, synaptogenesis, neurogenesis and gliogenesis via NPCs. In addition, Shh-induced profilin-1 involves in axon outgrowth, synaptic function, and neurotransmitter release. The Shh also confers neuroprotection by inhibiting PKA and activating PI3K. (B) Further, Wnt components have crucial impact on cognitive function via glucose metabolism and also provide synaptic strength by increasing PSD-95 expression. It can also help in neuroprotection and neuronal survival by increasing the expression of NGB, Norrin, GABA-R, NMDA-R, PPAR, and AchR. (C) Lastly, the Notch pathway imparts its major contribution in brain repairing and neuroprotection. For instance, it can help in maintaining mitochondrial biogenesis by increasing the level of TFAM and NRF-1. Further, Notch can also help in neuronal survival by reducing microbial activation, cytokines, apoptosis, and cerebral infarction. The neuroprotective action of Notch components is also mediated via NF-κβ signalling and NSCs. In this way, embryonic signaling can establish its neurotherapeutic potential in a range of neural disorders.

The therapeutic potential of bone marrow mesenchymal stem cell (BMSC) transplantation has been investigated in numerous brain injury models. Activated microglia-mediated neuroinflammation is the key hallmark of the pathogenesis of subarachnoid haemorrhage (SAH)-induced early brain injury (EBI). Interestingly, BMSC treatment mitigates the neurobehavioural impairments and inflammatory response in EBI post-SAH. Further analysis suggests that BMSC-mediated effects could be driven by Botch, a potent Notch antagonist that is upregulated in brain tissue post-trauma [320]. After ischaemic insults, the Neuroprotective gene 7 or Botch exerts neuroprotective effects by protecting the neurons, principally by antagonizing the maturation of Notch-1-triggered neuronal injury and neuroinflammation. In addition, Botch exerts neuroprotective effects by shortening neurobehavioural phenotypes, enhancing infiltration of activated microglia, improving inflammatory cytokine release and preventing neuronal cell death. Botch overexpression inhibited the generation of NICD and translocation of NICD into the nucleus, thereby preventing neuronal cell death by activating Notch downstream activators [321]. Further, we proposed pathways for the therapeutic intervention of embryonic signalling cascades in neurological disorders (figure 6). Finally, studies have comprehensively investigated the role of Notch in driving the neuroprotective role of microRNAs (miRNAs). A study reported that miR-98 binds to the Notch downstream target (Hey-2) and decreases the production of Aβ peptide and oxidative stress, and improves mitochondrial dynamics by activating the Notch signalling pathway in the mouse model of AD [84].

## 15. Conclusion

The embryonic signalling pathways play an essential role not only during normal embryonic development, as the name suggests, but also during adult neurogenesis. Therefore, it would not be incorrect to say that embryonic pathway footprints are present all across numerous processes, which are required to maintain normal brain homeostasis. In this review, we have summarized studies describing the association of deregulation of embryonic pathways such as the Notch, Shh and Wnt signalling pathways with neuronal development and neurological disorders. Currently, the knowledge of how embryonic pathways drive the aetiology of a panel of neurological abnormalities is limited compared with other human diseases. Hence, the findings of most of these studies are rather ambiguous in the sense that a lot remains to be understood and deciphered in terms of embryonic pathway components and their disease-causal interactions. Thus, this review has laid the foundation for gaining mechanistic insight into neurological abnormalities stemming from the deregulation of embryonic signalling. Eventually, the mechanistic insight into neurological abnormalities will prove beneficial for the future development of targeted therapies. However, the BBB should be considered when developing targeted therapies for neurological disorders because the BBB can restrict therapeutic agents that may otherwise be effective and result in poorer outcomes. Although there exists a converse argument that this hypothesis might not be essentially true, the role of embryonic pathways in maintaining BBB integrity is not disputed. Hence, adequate local drug concentrations should be achieved in many neurological diseases. In summary, the rapidly expanding interest in multifunctional drugs or biological compounds for the treatment of neurological disorders is creating new opportunities for the future development of novel neurotherapeutics. Thus, if dynamic prodromal diagnostic tools for the most common neurodegenerative diseases can be developed in parallel with the development of an array of multimodal drugs that target a reasonable selection of deregulated embryonic pathway cross-talks, significant strides will probably be made in the prevention and treatment of the most common and burdensome neurological disorders.

## Data Availability

This article has no additional data.
